# Is recycling sustainable: The promise and risks of scrap tire valorization

**DOI:** 10.1016/j.isci.2026.117462

**Published:** 2026-09-05

**Authors:** Mostafa Vahdatbin, Pouria Hajikarimi, Suliman Rashid, Elham H. Fini

**Affiliations:** 1 School of Chemical, Petroleum and Gas Engineering, Iran University of Science and Technology, Tehran 1684613114, Iran; 2 Department of Civil and Environmental Engineering, Amirkabir University of Technology (Tehran Polytechnic), Tehran 1591634311, Iran; 3 Arizona State University, 660 S. College Avenue, Tempe, AZ 85287-3005, USA

**Keywords:** sustainability, end-of-life tires, devulcanization, pyrolysis, circular economy, human health, environmental risk, microplastics, heavy metals, 6PPD

## Abstract

Approximately 1.5 billion end-of-life tires accumulate globally each year, presenting major environmental and human health challenges. Although recycling pathways such as devulcanization, pyrolysis, and mechanical grinding are promoted to conserve resources, their real-world sustainability is limited by technical, economic, and environmental constraints. These recycling processes can release hazardous pollutants, including volatile organic compounds, polycyclic aromatic hydrocarbons, heavy metals, microplastics, and 6PPD-quinone, posing long-term ecological and toxicological risks. Integrating technical, regulatory, and toxicological perspectives, this review evaluates the risks and benefits of current tire management strategies. It uncovers overlooked downstream impacts and calls for advanced recycling technologies, high-fidelity monitoring systems, and strengthened regulations to enhance the overall sustainability of tire recycling practices.

## Graphical abstract

**Figure:**
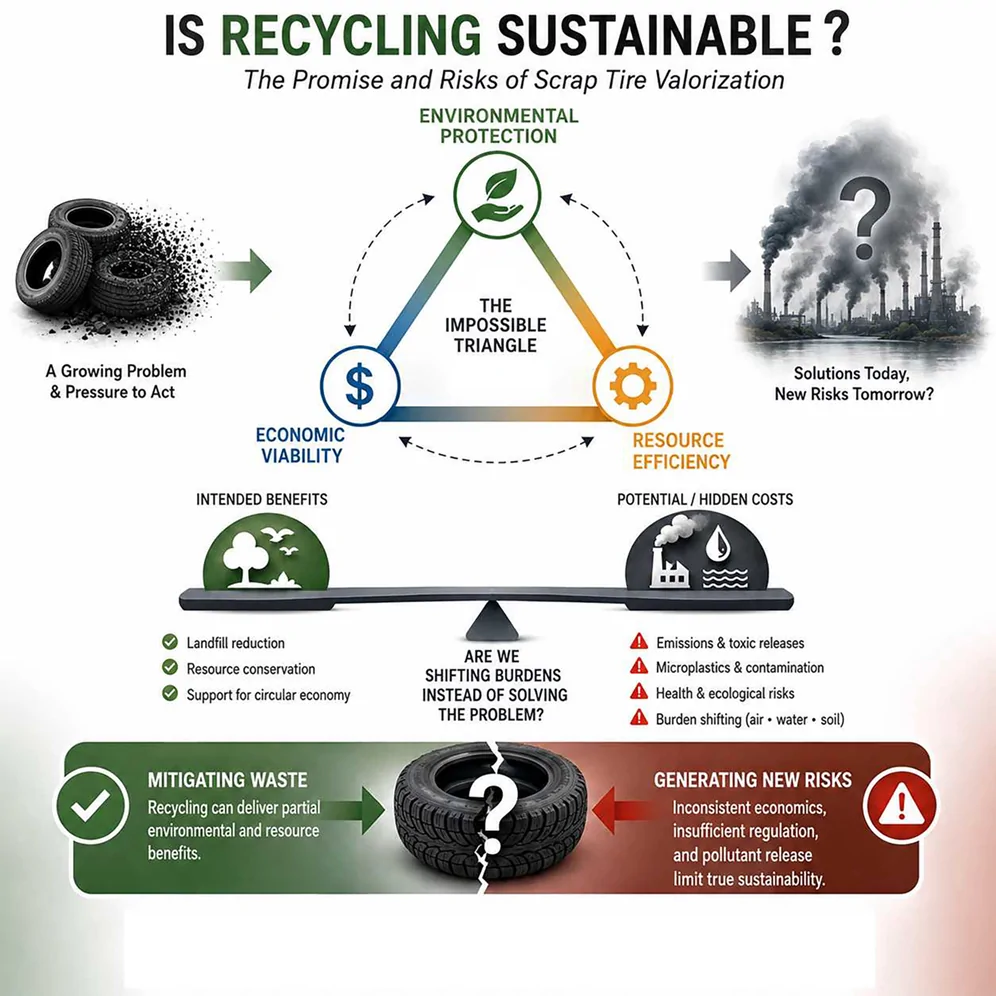

## Teaser

Environmental health; Environmental policy; Pollution

## Introduction

Socioeconomic development is manifested by the increasing number of vehicles worldwide. This has led to an increasing tire replacement frequency and, subsequently, steadily growing waste tire generation over the past few years. Tire rubbers are made up of elastomers, i.e., elastic polymers with high elastic memory and ultimate elongation but low elastic modulus.^1^ Tires are made of a complex mixture of highly engineered components, including belts, tread, sidewall, and inner liners. Each of these components has been designed so as to satisfy performance characteristics. Together, they form tires with high durability, strength, reliability, and safety, ensuring that tire constituents and materials persist in the environment.^2^ Waste tires represent a major, complicated type of waste produced worldwide, with an increasing production rate.^3^ They are non-biodegradable and can persist in the environment over a substantial period, posing a serious environmental challenge.^4^

The global tire market size is projected to reach about 2.7 billion units by 2028, with a compound annual growth rate of 2.8% from 2023 to 2028.^1^ Estimates suggest that as many as 5 billion tires will be discarded each year by 2030, particularly disposed of in landfills.^5^ Also, global waste generation is expected to almost double by 2050 and triple by 2100, compared to 2016.^6^

Tire tread possesses a complex chemical composition. The range of elastomer (typically in the following forms: styrene-butadiene rubber (SBR), natural rubber, or butadiene rubber) is around 30–60% by weight of the tire tread. Another 25–35 wt % is made up of inert fillers (e.g., silica and carbon black). The remaining mass is a blend of oils, plasticizers, protective molecules, and vulcanization agents. Around 1–3 wt % of tire tread comprises protective molecules (e.g., 2,2,4-Trimethyl-1,2-dihydroquinoline (TMQ) and N-(1,3-dimethylbutyl)-N′-phenyl-*p*-phenylenediamine (6PPD)), and 1–4 wt % consists of vulcanization agents (e.g., N-cyclohexyl-2-benzothiazolesulfenamide (CBS) and 1,3-diphenylguanidine (DPG)).^7^ Different tire layers have different chemical compositions (for example, winter tires differ from summer and all-season tires) and are significantly dependent on tire brands and applications. They also differ in terms of quantity and quality in different regions and countries.^8^ The natural-to-synthetic rubber ratio in tires is almost 50:50. For example, compared to truck tires containing more natural rubber, passenger car tires consist of a higher proportion of synthetic rubber. However, there is hardly any synthetic rubber in the tires of heavy-duty vehicles.^2^

**Figure 1:**
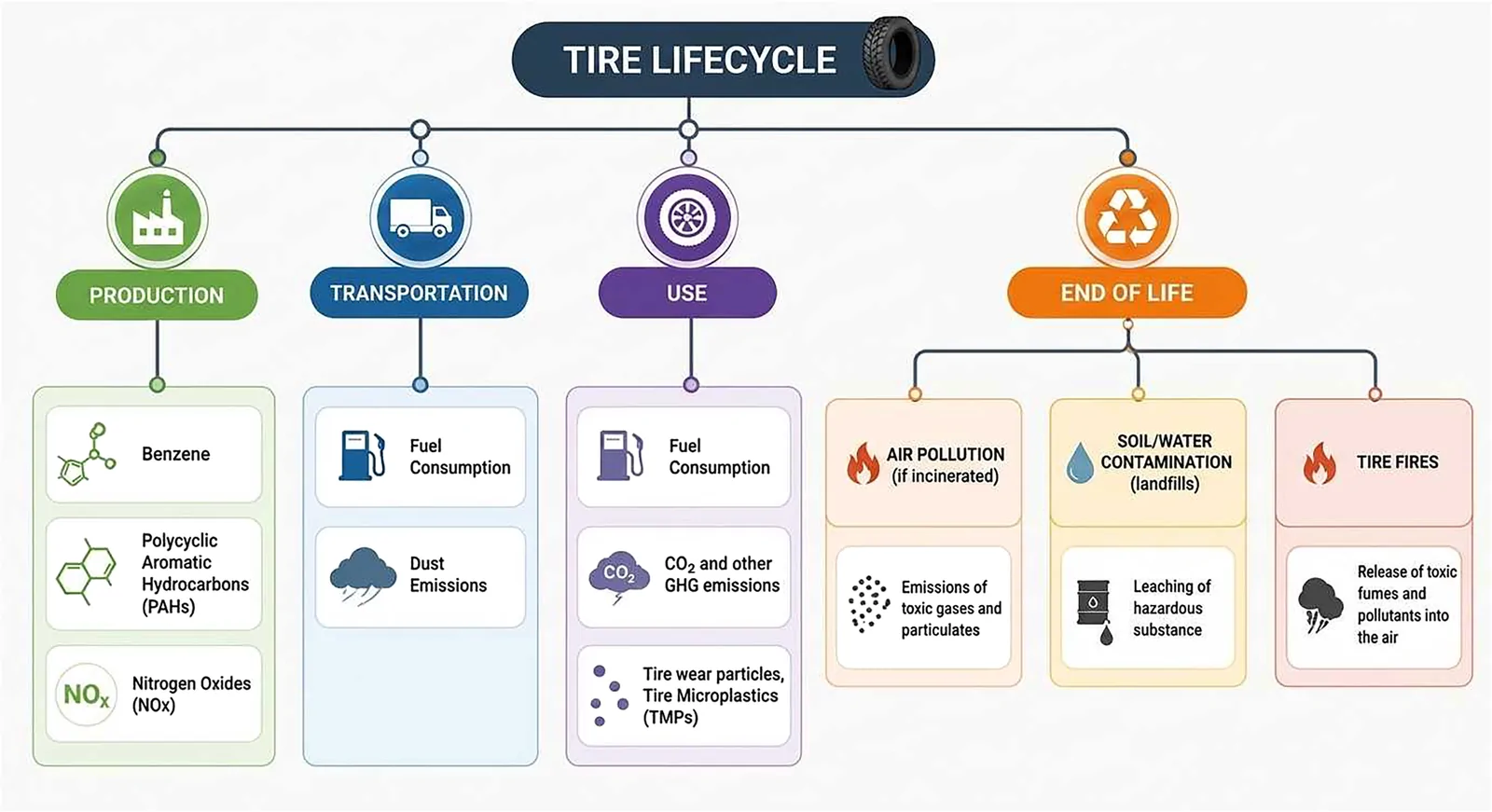
A hierarchical diagram illustrates the four main stages of a tire life cycle, along with major pollutants and outputs associated with each stage

There are four stages of a tire’s life cycle: production, transportation, use, and end of life. Each stage is associated with environmental threats and outputs, including pollutant emissions and resource consumption. These impacts are summarized in Figure 1, which presents a visual hierarchy of the tire life cycle and its associated environmental burdens.^9^

Energy consumption is the most prominent aspect of the environmental impact over the life cycle of tires on vehicles. Research has shown that energy accounts for nearly 96% of the carbon footprint and contributes 92% to the acidification potential. Material production has also been demonstrated to have major effects on a number of aspects of tires’ environmental impacts (e.g., a 92% impact on the human toxicity potential.^10^

Although the primary focus of this review is the end-of-life management and recycling of existing tires, with approximately 1.5 billion tires discarded annually worldwide,^11^ we briefly address tire-related pollution occurring during the use phase. Tire-road abrasion continuously generates tire wear particles, which are increasingly recognized as a significant yet often overlooked source of microplastic pollution. These particles can be transported through road dust, stormwater runoff, soil, aquatic systems, and atmospheric pathways, creating environmental burdens long before tires enter end-of-life management systems. Incorporating this use-phase perspective positions scrap tire recycling within a broader life cycle sustainability framework, where unintended environmental consequences may arise from tire formulation, in-service wear, particle release, and downstream recycling or valorization pathways.^2^^,^^8^^,^^12^^,^^13^^,^^14^

In terms of end-of-life tire recycling, it has been demonstrated that it would be highly complex to recycle cured rubber products because of the existence of a 3D sulfur cross-linked network structure arising from raw rubber vulcanization. In vulcanization, rubber turns into a thermoset after being crosslinked with sulfur. As opposed to thermoplastics, it is impossible to reform or restructure rubber into other products.^15^ Waste tires are non-biodegradable materials whose long-term open-air storage exhausts land resources and leads to serious environmental contamination. Since high-value utilization of waste tires has emerged as a critical global ecological problem, it would be challenging to find an affordable and harmless waste tire disposal method on a global scale.^5^ For sustainable development, global tire demand and huge quantities of recovered rubber must be met to develop appropriate end-of-life strategies and added value. Waste tires recovery rates are very high (*>*85%) in some regions and countries (e.g., Japan, the USA, and Europe), yet still very low in most others.^16^

Specific attention has been directed toward toxic emissions from tires during both their service life and end-of-life recycling, particularly volatile organic compounds (VOCs) and polycyclic aromatic hydrocarbons (PAHs). However, a comparatively underexamined yet highly relevant constituent is sulfur, which is intrinsically embedded in tire vulcanization chemistry. A typical new passenger car tire has a mass of nearly 8 kg, with a sulfur content of approximately 1.6 mass %, reflecting its essential role in forming and stabilizing crosslinked rubber networks. During service life, tire materials are continuously exposed to mechanical abrasion, elevated pavement temperatures, solar radiation, humidity, and oxidative stress, all of which promote gradual degradation of sulfur-containing additives and crosslinks. These processes result in the formation and release of both volatile and leachable sulfur species.^17^

In addition to end-of-life thermal and chemical recycling processes, real-world in-service conditions also contribute to continuous emissions of sulfur-containing volatile compounds. These include heterocyclic and organosulfur species such as thiazoles, thiophenes, benzothiazoles, sulfides, and mercaptans, which can be emitted directly from tire tread wear and indirectly formed through oxidative and photochemical transformation pathways.^18^ Notably, similar sulfur-derived emissions have also been identified from asphalt binders under elevated temperatures, solar aging, and moisture-assisted oxidation, where sulfur-containing heterocycles (e.g., thiophenes and thiazole-like compounds) and low-molecular-weight organosulfur compounds, some of which exhibit strong water affinity and high solubility, are released during binder degradation.^19^ These compounds contribute to localized air pollution, odor nuisance, and the formation of secondary atmospheric pollutants, while also entering aquatic systems via roadway runoff. Once released, many sulfur species exhibit persistence and mobility in environmental media and have been associated with adverse ecological and potential human health impacts.^20^

Incineration and landfilling or stockpiling of tires have caused numerous environmental problems. Stockpiled tires pose a significant fire risk, regularly leading to uncontrolled massive fires, demonstrating that these traditional solutions are inappropriate.^21^ On the other hand, incineration is the most polluting conventional solid fuel after coal in terms of greenhouse and toxic gas release and residual soot. Some popular green methods are recycling techniques with less energy consumption and greater recyclability of waste tires. In this context, manufactured product consumption rates are also a key factor, and products with low consumption rates will not exert a special environmental impact.^22^ The recycling of waste tires creates benefits for energy conservation as well as cost reduction and both raw material preservation and environmental protection.^23^ Thus, alternative solutions are required because tire waste is only partially reusable by the currently used tire waste recycling technologies. Accordingly, certain technical solutions must be sought for efficient tire waste recycling to mitigate environmental pollution and reach an efficient circular economy.^24^ Efficient rubber product circularity is secured by evaluating its major analytical tools, including recycling processes with large energy consumption that may cause environmental pollution, to avoid adverse effects. Thus, a sustainable circular economy necessitates conserving resources and reducing used tire deposits. Hence, certain technologies must be developed to effectively use secondary materials derived from used tires. In this context, such waste is more likely to be recoverable, and an efficient circular economy is ensured.^24^

Park et al.^25^ present a significant advancement by developing a tandem catalytic approach (combining isomerization and ring-closing metathesis) to convert end-of-life tire rubber into valuable chemical feedstocks. The study demonstrates advantages over conventional mechanical recycling and suggests a reduced environmental footprint, thereby contributing to the development of a more circular economy. Putro et al.^26^ developed a two-step recycling approach involving cyclodepolymerization of sulfur-crosslinked, carbon black–reinforced isoprene rubber via ring-closing metathesis, followed by pyrolytic conversion of the resulting oligomers into monomeric isoprene. The metathesis process enabled efficient solubilization and separation of the organic rubber components from carbon black under mild conditions. The recovered products consisted mainly of high-purity isoprene oligomers, predominantly cyclic tetramers, which were subsequently pyrolyzed to yield isoprene monomer and valuable aromatic compounds such as benzene, toluene, and xylene. However, concerns related to the potential health and toxicity impacts of such recycling processes remain insufficiently addressed. In this context, the present review highlights the associated health risks and emphasizes the need for comprehensive toxicological assessment and process monitoring to ensure the overall sustainability of emerging recycling strategies.

Several guidelines and standards have been published in the tire industry, particularly for end-of-life tires (ELTs). For example, in Europe, a series of directives have been released, including 1999/31/EU and 2000/53/EU to prohibit waste tire landfilling and prevent waste derived from vehicles and their components, respectively. The European Tire Recycling Association proposed an ELT management scheme that integrates tax models, producer responsibility, and the free market into a single framework. Several regulations have also been released for tire eco-labeling. For example, the *Industrial Standard Conditions for Comprehensive Utilization of Waste Tires* in Chinaoffer technological references to ELT recycling and retreading with environmental requirements.^9^

The key topics covered in this review outline the core aspects of scrap tire recycling, including the recycling process, its role in the circular economy, associated environmental and health risks, and the life cycle assessment (LCA) of recycled tires. The environmental and health risks discussed include PAHs and their toxicity, heavy metals, VOCs, airborne pollution, and sulfur emissions during recycling and reuse. Furthermore, the review addresses 6PPD and 6PPD-quinone (6PPD-Q), tire microplastics, and nano-carbon black.

## Results

### Means of tire recycling

Recycling has recently seen significant advancements with the development of new equipment and devices; however, its disadvantages highlight the need for finding more eco-friendly alternatives. Among the most notable developments are microwave-assisted devulcanization reactors, ultrasonic treatment systems, and continuous thermo-mechanical extrusion units such as twin-screw extruders.

The 19^th^ century is often referred to as the ‘rubber technology era' due to the fact that a broad spectrum of challenges was tackled through rubber technology during that period. The recycling of rubber and its derivatives has advantages and disadvantages, as with other technologies. A review of the 20^th^-century records can reveal major advances in the management and processing of waste rubber in machine manufacturing and operation.^27^ Increasing attention is now being drawn to high-value recycled rubber products. Generally, there are three main directions for waste tire recycling, including ground tire rubber (GTR), reacted and activated rubber (RAR), and liquefied rubber (LR).^28^ GTR involves mechanically grinding tires for use in various products like asphalt, concrete, or polymers. RAR refers to rubber that has been devulcanized, treated, and functionalized to facilitate reuse. LR is obtained through thermomechanical processes, such as pyrolysis, which break down tires into useful products like fuels and chemicals.

In grinding, certain technologies are adopted to mechanically reduce the size of waste rubber components (e.g., ambient, cryogenic, and wet grinding) and produce a varying-size rubber powder that has several applications and serves as the starting operation for rubber devulcanization.^29^ Ambient grinding involves mechanically breaking down the vulcanized rubber at room temperature, a usually simple and cost-effective process, which can become very costly in case of a very fine mesh size. Also, the crumb rubber (CR) may be degraded by milling-induced heat generated in large quantities.^30^ In cryogenic grinding, liquid nitrogen is initially used to freeze the rubber, and subsequently, the frozen rubber is crushed using an impact mill. This is a cleaner, slightly faster process that results in the production of finer rubber crumbs. Ambient and cryogenically ground CR have different morphologies and surface structures. The latter has a relatively smooth surface and a broad range of particle size distribution. A cryogenic process plant entails higher operating costs than the ambient process due to the presence of liquid nitrogen and an additional drying step taken for moisture removal from the CR.^31^ This process produces rubber particles smaller than 2 mm, utilizing water as both a cooling agent and lubricant during the mechanical reduction phase. Also, a water jet is used in this process to mill large, highly resistant tires from trucks, leading to high-purity crumbs with a high specific surface area. Therefore, this is an environmentally benign process because of its low energy consumption and water recycling by means of a closed-loop system. Finally, the ground products must be dried.^15^

Apart from traditional cryogenic and ambient grinding techniques, waste rubber can be ground through the solution grinding, elastic deformation grinding, and ozone cracking grinding techniques. Such procedures downsize rubber particles in order to maximize their specific surface area for further procedures, such as re-vulcanization and mixing GTR in a powder or particle form with thermoplastics. To perform solution grinding or wet grinding, it is required to expose the waste rubber to fatty acids or solvents so that it swells before it is meshed and placed in conventional lines to be pulverized. Finer rubber particles can be obtained by combining cryogenic or ambient grinding with solvents. An ozone cracking setup applies a high ozone concentration to waste rubber to break it down into finer particles before the rubber is mechanically pulverized. However, ozone oxidation due to the pulverization process reduces surface reactivity in ozone cracking grinding. Nevertheless, ozone grinding is a green and eco-friendly technique. It promotes the scission of polymer cross-links, which aids in the breakdown of polymer chains and facilitates the thermal degradation of GTR. The elastic deformation grinding process is performed by mixing GTR and thermoplastics at a high shear force and pressure within a Banbury mixer. Then, a single- or twin-screw extruder is employed to grind the mixture. The advantages of elastic deformation grinding include utilizing cooling to melt the thermoplastic at a low temperature and the potential integration with other grinding methods.^27^

GTR is used to appropriately modify polymers as a promising approach to developing novel products for construction/building materials or the automotive industry. Several attempts have already been made in this field; GTR has been effectively applied during manufacturing, as in brake pads, roofing tiles, floating trays and buoys, 3D printed materials, tires, and ballast mats for railways. However, polymer composites have a usually limited GTR content.^28^ Given that the use of CR obtained from waste tires is an effective solution to the environmental problems resulting from the disposal of end-of-life tires, its use in the production of asphalt pavement has received significant attention recently.^32^ In order to recycle waste tires for the production of asphalt pavement, two techniques have been used: the dry and wet processes.^33^ While during the dry process, the fine aggregate in the asphalt mixture is replaced by CR, it is added to the hot asphalt binder as a modifier in the wet process in order to create the modified binder, which is then blended with aggregates.^34^

Devulcanization is a recycling procedure in which crosslinked rubber chain bonds are cleaved. It is a process during which rubber waste is transformed into its original uncured form by selectively breaking covalent cross-links in an elastomeric matrix. In theory, the devulcanization yield would be a blend of the prepared uncured rubber for vulcanization with intact polymeric chains. Devulcanization has a long history and has attracted considerable attention from researchers, especially over the last decade because public concern has arisen regarding waste rubber management.^35^ Numerous rubber recycling methods have been previously documented in the scientific literature. Particular focus, in order of frequency of mention in the literature, has been placed on mechanical, microwave, chemical, integrated thermo-chemo-mechanical and microwave, thermo-chemo-mechanical, ultrasonic, microbial, and cryogenic approaches. This highlights the application of sustainable methods and technologies.^1^ Flowability (similar to thermoplastics) and re-vulcanizability represent key requirements, regardless of the devulcanization process; the former allows for producing new products, while the latter ensures that a useful product can be produced.^36^

During the pyrolysis process, waste tires are thermally decomposed at 400°C within an inert environment without oxygen. It has three byproducts: tire pyrolysis oil (TPO) (40–60 wt %), pyro-gas (5–20 wt %), and pyro-char (30–40 wt %). TPO is used to synthesize carbon nanotubes. The gross heating value of pyro-gas is in the 30–40 MJ Nm^−3^ range. A gaseous fraction (comprising non-condensable gases such as H_2_, CO, CO_2_, C_2_H_4_, C_3_H_6_, and so on) is utilized as fuel during pyrolysis. Pyrochar has extremely high carbon content, rendering it applicable for making porous activated carbon adopted for studies on the adsorption of harmful gases.^37^ A recent study has explored its use in energy storage applications, such as Li/Na/K-ion batteries, supercapacitors, and catalysts for biodiesel production.^38^

### Circular economy implications of tire recycling

As an economic system, while decreasing waste generation, the circular economy values resources by keeping materials in use for longer periods. By contrast, a linear economy functions on the basis of a ‘take, make, and dispose’ model.^6^^,^^39^ Through the prioritization of prolonged circulation of materials and using technologies and products to minimize environmental impacts and waste, the circular economy challenges the linear economy model. Through the provision of a comprehensive solution to environmental concerns and promotion of sustainable development, such a strategy goes beyond economic planning.^39^

One of the novel study fields for the further enhancement of the economic advantages of the process is the recycling of technical products in order to produce new tires. At the same time, the closed-loop concept is in agreement with the aim of minimizing resource inputs sought by the circular economy.^40^ Besides providing long-term social, economic, and environmental advantages, resorting to a circular economy preserves natural resources and creates new jobs. For instance, an estimate has demonstrated that recycling every 10,000 tons of waste creates 9.2 jobs; however, this figure is only 2.8 for landfill disposal.^6^ In 2020, a $600 million fund was allocated to waste recycling by the Australian industry and government, with an expectation of creating approximately 10,000 new jobs.^41^ Also, a circular economy in the European Union is estimated to result in a €600 billion saving while creating 580,000 jobs and decreasing 450 million tons of carbon emissions by 2030.^6^

Finding technical solutions allowing efficient recycling of used tire waste is necessary in order to decrease environmental pollution and reach an efficient circular economy. In order to make the circularity of rubber products more efficient, it is required to evaluate its primary analysis tools so as to avoid unfavorable effects. For instance, it is necessary to evaluate the recycling processes featuring large energy consumption, which may turn into a polluting source for the environment. As a result, conservation of resources and reducing used-tire deposits is necessary in order to ensure a sustainable circular economy. Therefore, finding novel technologies ensuring superior utilization of secondary materials from used tires is necessary to increase the chance of the recovery of these wastes and ensure an efficient circular economy.^24^

Given that the granulation of used tires necessitates advanced processing and treatment steps, in general, the investments required for granulation and its applications can be significantly higher in comparison with the other recycling techniques. Moreover, a number of markets for ground rubber applications, e.g., its utilization in artificial sports fields, have already decreased as a result of continued debates about the carcinogenic materials potentially released in the course of tire production. Recent measures taken by the European Commission, e.g., the restricted use of microplastics intentionally added to products, will further hinder this application. Since granulation is regarded as a material recycling process, it is high up in the rubber waste management hierarchy. Nonetheless, the aforementioned uncertainty about the potential environmental and health hazards related to a number of applications of rubber granules will most likely impose more limitations on such a recovery technique in the future.^42^

Through reduced resource consumption and alleviation of environmental pollution, the incorporation of scrap tire rubber into asphalt paving mixtures may play a key role in the circular economy. Nonetheless, their use is a part of long-continued discussions about the safety of CR and the potential release of carcinogenic materials utilized in the tire manufacturing process.^43^^,^^44^^,^^45^ Although retreading is consistent with the circular economy and, in order to increase the service life of products, retreading functions as an established practice only for aircraft and truck tires at present; due to economic profitability, it is not used for automobile tires. Besides the small market of civil engineering applications for shredded or whole tires, such applications generally present economic benefits over conventional substances.^42^

As an encouraging pathway in rubber recycling, devulcanization encompasses a collection of technologies specifically targeted at rupturing the sulfur cross-links in vulcanized rubber while the damage inflicted on carbon-based molecular rubber polymer chains becomes minimal. This paves the way for the reversal of the rubber vulcanization process, which makes the substance favorable to add to rubber compounds when producing new products. Thus, devulcanization is regarded as one of the key technologies to enable the shift toward a circular economy for tires. Even though devulcanization technology has not been implemented on an industrial scale yet, once the markets shift toward the circular economy, this can be changed. A global evaluation was carried out in 2004 to study the use of devulcanization for recycling tires. Devulcanization was conducted only for development and research purposes at a maximum scale of 45 kg/h. At that time, the scholars claimed that devulcanization is contested to remain competitive against virgin rubber. The asserted limitations included no clear standards, the absence of reliable data on devulcanization, and short-term, price-driven markets.^46^ Nonetheless, since that time, devulcanization has been progressively developed, and currently, it is extensively being applied by various companies around the world.^47^

Even though a great number of technical innovations have occurred to push devulcanization,^48^ a shift toward a circular economy necessitates systemic innovations. These are exhaustive innovation processes that change materials and products flow through value chains in the course of their lifespan.^49^ Nonetheless, the main body of academic literature on devulcanization is mainly focused on the technical dimensions. Nonetheless, value chains are critical because the industrial devulcanization process involves different process steps, such as separating tire rubber into powder or granulate, sorting tires, applying devulcanized material in new products, and conducting the devulcanization process. The development of these value chains is an innovation process in which organizations encounter a variety of constraints and opportunities. A knowledge gap apparently exists regarding the use of industrial devulcanization technology so as to innovate tire recycling in value chains.^46^

All the materials produced through the pyrolysis process, such as oil, syngas, high-quality carbon black, and residual steel, can be used for different purposes. Nonetheless, at present, the economic viability of pyrolysis is still quite low compared to virgin or conventional materials. Nonetheless, given the ever-increasing gas and oil prices and the increasing level of technical feasibility, pyrolysis can become more important very soon. In fact, some European companies are trying to commercialize the development of tire-derived material carbon black. Given that, in the case of ignoring environmental standards, pyrolysis may result in noticeable air pollution from an environmental viewpoint, high environmental standards along with continuous monitoring of emissions are necessary when developing the pyrolysis technology further.^42^

### Environmental and health risks of tire recycling

While tire particles show relatively high persistence in the environment, they can be easily affected by biotransformation, phototransformation, hydrolysis, mechanical degradation, and oxidation-reduction (redox) processes.^2^ They are also subjected to thermal degradation and can sometimes reach 60°C or higher on hot asphalt.^50^ Tire rubber is sensitive to ozonolysis, i.e., oxidatively cleaving C=C double bonds, resulting in rubber cracking and crazing. Considering its durability, the physical modification of tire rubber should be fairly limited over short-term time scales, except for mechanical degradation. However, tire particles are likely to agglomerate with other particles present in the environment.^2^ The release of toxic aromatic compounds from tires into the environment is a serious problem since recycled tires are extensively used in a variety of applications^51^ where there is direct contact between humans and these materials. This is because tire constituents may be leached and/or volatilized into the environment over time.^52^ Several studies have analyzed the concentrations of aromatic compounds in air, soil, and leachate depending on various applications of scrap tires, including artificial turf fields, asphalt, playgrounds, etc. These compounds have been frequently shown to be present in leachate from scrap-tire-shreds or artificial turf sports fields.^51^^,^^53^^,^^54^

Whole tires can endure under various environmental conditions. However, CR is produced by reducing tires down to sizes ranging from 20 to 80 mesh particles. The larger surface area of smaller CR particles is exposed to various environmental conditions, thereby accelerating the release of tire constituents into the environment. Several mechanisms may initiate and accelerate this process, including ozone reaction with rubber surface to form cracks, oxygen-induced rubber oxidation, water-driven leaching of soluble chemicals, heat-induced acceleration of CR oxidation, sunlight-mediated acceleration of CR oxidation and degradation of surface anti-degradants, wet climate-promoted loss and leaching of protective coatings while hot; dry climate-induced oxidation, and weather conditions (e.g., freezing) facilitating rubber cracking.^52^

Despite its application, rubber interacts with the environment and releases hazardous materials into the groundwater and soil. Consequently, a thorough examination must be conducted on leachate to consider and replicate the conditions potentially facing waste rubber products. Waste tire rubber recycling helps partially solve the currently increasing pollution problem and offers innumerable advantages to civil engineering materials. Nevertheless, the potentially adverse health and environmental impacts connected to this process must be recognized and addressed. This necessitates conducting critical environmental studies, including a thorough examination of leachate analysis to ensure that the proposed waste tire recycling method indeed reduces its ecological footprint.^39^ Moreover, economic efforts should be made to activate waste tire recycling procedures for environmental conservation to offer productive recycling opportunities. Nonetheless, special attention is now devoted to waste characteristics and applications. It is still significantly challenging to find a sustainable waste tire recycling pathway, where interconnected and complex economic and environmental issues should be dealt with. Thus, policymakers must be provided with precise evaluations and accurate descriptions of waste utilization from various perspectives, including economic costs, optimization directions, and environmental impacts.^55^ According to literature data, besides the ingredients above, recycled rubber granules applied in playgrounds and sports fields may contain chemicals toxic to the environment and/or human health. They may originate from poor-quality raw materials or impurities in vulcanized rubbers. They contain PAHs, phthalates, VOCs, and heavy metals (e.g., Pb, Cd, As, and Hg).^53^ Each contaminant may have varying concentrations based on the age and basic composition of the parent material utilized in the turf and infill.^44^ There are several major routes through which hazardous substances may enter the human body, including alimentary, inhalation, and direct skin contact.^53^

Contrary to the desirable rheological characteristics of asphalt rubber (AR), the recycled CR derived from waste tires includes impurities causing a potential threat to the environment when asphalt rubber is utilized as a paving material. Tire crumb rubber may release toxic pollutants, e.g., heavy metals, including zinc (Zn) and PAHs, which may leach from CR-modified asphalt pavements, causing environmental pollution.^34^ For instance, stormwater runoff may transfer pollutants, such as PAHs, VOCs, and metals contained in road pavement materials to the groundwater. The infiltration of rainwater through overlying soils or the direct contact of materials with surface water or groundwater may constitute the origin of the contacting water. The contaminants leaching into the water may lead to further contamination of the neighboring soils or disperse into surface water bodies or groundwater.^56^

The chemical compositions of rubber modified asphalt (RMA) demonstrate distinct patterns from those of regular asphalt, which affects their impact on the environment. Li et al.^57^ document that RMA thermal processing emits volatile compounds consisting of VOCs and H_2_S and various PAHs, among which PAHs demonstrate significant contributions toward ozone formation potential (OFP) and secondary organic aerosol formation potential (SOAFP). CR increases both the quantity and toxicity of compounds that are released during heating, especially during the initial heating period. According to Mousavi et al.,^58^ regular asphalt prepared at 150 °C over 24 h generated emissions that mostly contained single-ring aromatic compounds, alkanes, and alkenes but none of the main volatile organic compounds included PAHs. The regular asphalt study demonstrated effective VOC reduction by adding biochar because it reduced environmental burdens from PAH emissions, which suggests better potential for pollution control.

The research conducted by Li et al.^57^ demonstrates that RMA heating at 180 ± 2 °C affects both the fume composition and their emission responses. Research shows that H_2_S emissions appeared mainly during the first 150 min, and VOCs dominated emissions from 0 to 270 min. Under high-temperature and oxygen-depleted conditions, alkanes within fumes easily participate in dehydrogenation reactions to generate alkenes with corresponding carbon structures. When exposed to thermal oxidation, alkanes experience changes, and the process produces alkynes and aldehydes along with ketones and esters. Aromatics that have been hydrogenated will evolve into PAHs. The exposure time duration affects hydrocarbon derivatives (HYD) more than aliphatic hydrocarbons (ALH) during prolonged thermal exposure, thus demonstrating an ongoing trend of increase. Thermal treatment of small-sized CR in modified asphalt caused the concentration of 13-docosenamide to become the most dominant compound after 270 min of exposure. The experimental findings show that RMA releases PAHs and high-weight ALHs when subjected to thermal treatments due to their fundamental role in ozone formation and secondary organic aerosol production.

Nonetheless, most of the earlier studies have concentrated on the improvement of the compatibility between CR and asphalt binder and the optimization of the engineering performances of the asphalt rubber produced.^59^^,^^60^^,^^61^ Also, limited investigations have been carried out to study the potential leaching characteristics of asphalt rubber. The potential risks resulting from the utilization of asphalt rubber have not been scrutinized adequately, and the amount of chemical constituents leaching from asphalt rubber is not clear yet. In addition, earlier investigations have not addressed the effect of inevitable aging on the leaching characteristics of asphalt rubber. As a result, it is vital to conduct an in-depth study of the factors contributing to the leaching of these chemicals and a fundamental understanding of the potential leaching risks of asphalt rubber subject to real-world conditions.^34^

Material recovery is another commonly used tire valorization method. Particularly, the final tire crumb is crumbed and reused as infill in compounds for roads, playground surfaces, and sports. Europe is currently using approximately every third of ELT for this recycling route. For example, scrap tire rubber can be included in asphalt paving mixes through wet and dry processes. During the wet process, the general function of CR is to modify the asphalt cement modifier to enhance the asphalt binder’s rheological properties. Conversely, during the dry process, CR helps modify the bitumen or partially replaces aggregates. Both processes significantly contribute to the circular economy by minimizing resource consumption and mitigating environmental pollution. However, there is a long-standing discussion about their application regarding CR safety and the possible release of carcinogens (e.g., antioxidants, antiozonants, and PAHs) into the environment during tire manufacturing. Over the past decade, several studies have demonstrated that CR infill does not pose any threat to human health. Nevertheless, as this remains an issue of concern and microplastic classification includes CR, the EU may inevitably restrict or even completely ban this application. Indeed, following lengthy negotiations, the EC and EU member states decided to place a ban on the use of mulches and granules as infill if they contained more than 20 mg/kg of the sum of PAHs to ensure safer playing on playgrounds and artificial sports pitches.^30^

Most patented reclaimed-rubber technologies rely on thermo-chemical dynamic desulphurization in specialized reactors because it produces superior mechanical properties compared to alternative methods. The process operates under high temperature and pressure with water and requires additives such as activators and softeners, whose selection strongly influences material quality. Despite its industrial practicality, the method is associated with odor, pollution, high energy and water consumption, and significant emissions.^47^

Devulcanization of byproducts (e.g., H_2_S, SO_2_, and CS_2_) produces environmental and health hazards like toxic gases, thus necessitating further treatment. There are several devulcanizing chemical agents, including disulfides (diphenyl disulfide and thiophenols), thiol-amine reagents, and hydroxide or chlorinated hydrocarbons, which share similar radical-scavenging activity to prevent re-crosslinking during devulcanization. Generally, while these substances have typical effective concentrations in the 0.5–10 wt % range with respect to GTR, they are often high-priced, and their uncontrolled release may present serious long-lasting environmental issues. Some of these environmental concerns can be alleviated by the use of tunable, biodegradable, and eco-friendly devulcanizing chemical nd bio-treatment agents.^22^ Chemical reclamation techniques exhibit high desulphurization efficiency. Besides, numerous devulcanizing reagents are available, most of which are environmentally unfriendly and may pose odor problems with finished products that incorporate chemically reclaimed CR. Hence, non-sulfur compounds are now recognized as greener alternatives. Not only may reclaimed rubber be highly environmentally beneficial but it can also pose environmental challenges and hazards. Essentially, chemical additives (e.g., accelerators, plasticizers, and vulcanizing agents) should be utilized in certain processes of reclaimed rubber production, thereby causing environmental risks if they are not well managed. However, any reclamation technology and the final rubber products must undergo a comprehensive life cycle assessment to vividly depict the economic and environmental benefits for each individual case. Unfortunately, there is still extremely limited robust data for this purpose.^30^ During ELT pyrolysis, numerous environmental problems arise as a result of converting accelerators with various compositions into polar aromatics. Accelerators refer to N- and S-containing polar organic compounds that are either released intact during pyrolysis or converted mostly into N- or S-polar aromatics.^22^ There are different possible applications for all pyrolysis products (e.g., high-quality carbon black (CB), synthesis gas, oil, and residual steel). Pyrolysis now has relatively low economic viability with respect to traditional or virgin materials. However, pyrolysis may soon assume greater importance with rising oil and gas prices and technical feasibility levels. In fact, several European companies are now especially commercializing the tire-derived CB development. Nevertheless, from an environmental standpoint, pyrolysis can significantly contribute to air pollution if environmental standards are not met. Thus, it is crucial to advocate environmental standards while continuously monitoring emissions in the process of further development of this technology.^30^

Table 1 depicts different scrap tire recycling techniques, including their main process or product, circularity potential, energy use, emissions profile, scalability, and technology readiness level (TRL). Table 2 provides an overview of the potential environmental impacts associated with each recycling technique, highlighting concerns such as toxic emissions, hazardous byproducts, and pollution risks.

**Table 1:** Comparative assessment of scrap tire recycling techniques, including sustainability indicators, scalability/industrial maturity, and technology readiness level (TRL)

Recycling Technique	Main Process/Product	Circularity Potential	Energy Use	Emission Profile	Scalability/Industrial Maturity	Approximate TRL
Dynamic desulphurization	thermo-chemical devulcanization of vulcanized rubber under heat, pressure, and additives	high	high	high VOCs, wastewater, odor, chemical emissions	commercially established in Asia	8–9
Microwave treatment	microwave-assisted devulcanization of ground tire rubber (GTR)	high	moderate	lower chemical emissions than thermo-chemical methods	pilot to semi-industrial scale	5–7
Chemical methods	use of solvents, oils, disulfides, or chemical agents to break sulfur crosslinks	moderate to high	moderate to high	potentially hazardous chemical residues and solvent emissions	pilot to industrial depending on reagent system	5–8
Mechanical devulcanization	shear-induced bond scission using extrusion, grinding, or milling	moderate	moderate	relatively low direct emissions	industrially feasible and scalable	7–8
Biological devulcanization	microbial or enzymatic sulfur bond cleavage	very high	low	very low emissions and chemical demand	mostly laboratory scale	2–4
Reclamation	combined thermal, mechanical, and chemical recovery of rubber for secondary applications	high	moderate to high	moderate emissions depending on additives	mature industrial process	8–9
Combustion	energy recovery through direct combustion	low	net energy producer	high CO_2_, NOx, SOx, particulate emissions	fully commercialized globally	9
Material recovery/size reduction	mechanical shredding and grinding into crumb rubber or GTR	moderate	moderate	low to moderate particulate emissions	highly commercialized	9
Retreading of old tires	reuse of tire carcass with new tread	very high	low	significantly lower emissions than new tire production	fully industrialized worldwide	9
Pyrolysis	thermal decomposition under oxygen-free conditions producing oil, gas, char, and steel	Moderate	high	moderate to high depending on gas cleaning	commercial and rapidly expanding	7–9

**Table 2:** Potential environmental impacts of various scrap tire recycling approaches

Recycling Technique	Potential Environmental Impacts	Reference
Dynamic desulphurization	auxiliary agents (e.g., softeners, activators, aromatic oils, pentachlorothiophenol, ferric chloride, diethylene glycol diethyl ether, etc.) show heavy pollution emissions throughout the process due to the release of toxic byproducts.	Wiśniewska et al.^47^
Microwave treatment	volatile degradation products are emitted into the atmosphere.	Wiśniewska et al.^47^
Chemical methods	the chemicals utilized during the process can be toxic, potentially causing environmental issues if not properly managed. The improper valorization or handling of these chemicals can lead to environmental contamination.	Chittella et al.^29^
Mechanical devulcanization	hazardous gases (CS_2_, SO_2_, and H_2_S) are generated as byproducts.	Kumar et al.^15^
Biological devulcanization	the biological or bacteriological contamination from the by-products may pose risks.	Kumar et al.^15^
Reclamation	the utilization of potentially toxic chemicals and/or substantial heat; Severe secondary pollution.	Wu et al.,^40^ Goevert^42^
Combustion	release PAHs during the process; Highly toxic tire fire smoke can worsen air quality, leading to the pollution of extremely toxic gases such as CO_2,_ CO, SO_x_, NO_x_, and dioxins, while also reducing visibility in the vicinity.	Wang et al.,^5^ Kumar et al.^15^
Material recovery/size reduction	carcinogens may be released into the environment.	Dorigato et al.^30^
Retreading of old tires	this process presents major safety concerns for workers due to the off-gassing of toxic compounds such as VOCs, which can pose significant health risks.	Kumar et al.^15^
Pyrolysis	the process can significantly contribute to air pollution.	Dorigato et al.^30^

Accordingly, severe health, environmental, and safety problems may arise if they are not properly managed. Artificial turf manufacturers claim their products are harmless to public health and offer an efficient used tire recycling method.^51^ However, such claims have been challenged in several studies demonstrating the existence of certain pollutants that potentially cause human health and environmental problems.^11^^,^^62^^,^^63^ Notwithstanding, disagreements remain over such compounds and their potential role in health. In addition, since researchers have failed to reach a consensus on the existing data and the relevant knowledge gap, some environmental groups and recycled product users are unsure about their use and daily exposure to their released compounds. Given these opposing studies on human health risks, a comprehensive study should be carried out on the health and environmental impacts of using recycled tires. This way, policymakers can make sound decisions about regulations formulated on using recycled tires as they are supplied with a wealth of information.^51^

During tire degradation and recycling processes, toxic compounds, such as PAHs, VOCs, heavy metals, nano-CB, TMPs, and 6PPD along with its transformation product (6PPD-Q) are released into the environment. These contaminants originate from various sources and exhibit environmental persistence, posing significant risks to ecosystems, wildlife, and human health. Table 3 summarizes these toxic compounds, their sources, environmental impacts, and associated health risks.

**Table 3:** Toxic compounds in tires and their environmental and health impacts

Toxic Compound	Source	Environmental Impacts	Health Impacts	Specific Diseases/Effects	Reference
PAHs	process oils, CB, and extender oils in tires	leach into biofluids, soil, water persist in the environment; toxic to aquatic life	carcinogenic, mutagenic, immune toxic, teratogenic; bioaccumulation in adipose tissues	tumors in breasts, bladder, esophagus, colon, pancreas, skin, and lung; reproductive system abnormalities	Mayer et al.,^2^ Zou et al.,^34^ Sibeko et al.,^52^ Grynkiewicz-Bylina et al.,^53^ Pochron et al.^64^
Heavy metals	tire manufacturing (activators, pigments)	toxic to soil organisms (e.g., earthworms); leach into water and soil; impact microbial activity	nervous system damage; bioaccumulation; organ toxicity.	reduced fertility, decreased body weight, Zn toxicity to invertebrates and plants.	Mayer et al.,^2^ Zou et al.,^34^ Grynkiewicz-Bylina et al.,^53^ Pochron et al.,^64^ Sheng et al.^68^
VOCs	rubber degradation, devulcanization	airborne and water pollution; form new harmful chemical structures during degradation	respiratory issues; genotoxic effects	increased earthworm mortality; potential chronic toxicity from inhalation	Ruffino et al.,^54^ Pochron et al.,^64^ Formela,^73^ Azizian et al.,^71^ Hejna et al.^79^
6PPD and 6PPD-Q	antiozonant in tire rubber; released during tire wear; recycling, and from CR-modified asphalt	highly persistent in soil, water, and air; widespread in road runoff, dust, and stormwater; toxic to aquatic organisms (e.g., coho salmon, brook trout, zebrafish); environmentally stable; accumulates rapidly in aquatic media; causes ecological imbalance;	human exposure via inhalation, ingestion (e.g., fish), skin contact, and indoor/outdoor dust; detected in urine, including in pregnant women and children	fish mortality (coho salmon, brook trout, steelhead); liver toxicity; potential human toxicity (bioaccumulation); concern for pregnant women and children; possible long-term organ damage;	Johannessen et al.,^95^ Varshney et al.,^94^ Helm et al.,^100^ Papan,^102^ Zhao et al.,^103^ Zoroufchi Benis et al.,^104^ Ji et al.,^105^ Song et al.,^106^ Montgomery et al.,^107^ Grasse et al.,^108^ Peng et al.,^109^ Lokesh et al.,^110^ Scholz et al.,^97^ Wagner et al.,^111^ Jiang et al.,^112^ Bhalode et al.,^113^ Luo et al.,^131^ Jiang et al.,^98^ Chen et al.,^101^ Du et al.,^92^ Chung et al.,^91^ Hu et al.,^93^ Chang et al.,^96^ Deng et al.^99^
TMPs	tire wear particles, recycled tire crumb, tire repair-polished debris	persist in ecosystems; second-largest contributor to marine microplastic pollution	health risks through air, water, and food chain exposure	bioaccumulation, unknown specific diseases	Luo et al.^8^
Nano-Carbon Black	fillers in tire rubber; bound to tire wear particles	released into the environment through weathering and abrasion; contribute to nanoparticle pollution	potential respiratory and systemic impacts from nanoparticle exposure	unknown specific diseases; fragmented nanoparticles through wear	Kim et al.,^117^ Lin and Wang^132^

### PAHs and their toxicity

Among the potentially toxic organic compounds in CR, PAHs, present in process oils, are frequently regarded as extremely hazardous to human health. They consist of 2–6 fused aromatic rings made up of solely hydrogen and carbon atoms. They range in appearance mostly from colorless to white or pale yellow-green and exist in over 100 different combinations.^52^ They may originate from CB and extender oils utilized for tire manufacturing in SBR granules.^53^

Tires contain varying types and quantities of PAHs. They leach from tire particles and other tire debris in biofluids, soil, and water. They also have high environmental persistence or can be transformed into other potentially toxic compounds. Besides, while they account for most of the toxicity of tire leachates and debris exposures, their proportion in settings originating from tires is unclear. There is a similarity between observed PAH exposure-induced abnormalities and those observed in tire debris-exposed fish. PAHs, along with their metabolites, can have both carcinogenic and non-carcinogenic effects. For example, tire particle-derived PAH uptake may lead to mortality and developmental defects in fish. Also, PAH exposure may result in cardiotoxicity and immunotoxicity and disrupt reproduction in wildlife.^2^ Exposure to PAHs, which may take place via dermal contact or water ingestion, may result in many adverse effects. A number of PAHs are ecotoxic and exhibit carcinogenic, mutagenic, immunotoxic, or teratogenic effects on living organisms. Given their high lipophilicity, PAHs tend to bioaccumulate in adipose tissues. Long-term exposure to PAHs may result in the formation of tumors in some organs, e.g., the breasts, bladder, esophagus, colon, pancreas, skin, and lung. Furthermore, PAHs are characterized by embryotoxicity, and, given their adverse effects on testicular function, sperm quality, egg viability, and nucleic acid damage, they are associated with reproductive system abnormalities.^34^

Generally, atmospheric PAH concentrations above outdoor artificial turf fields were similar to local PAHs within limits set by regulations.^51^ Low-dose PAH exposure can cause impaired metabolic and immune system functions as well as genotoxic damage to earthworms.^64^ Lu et al.^65^ concluded that the artificial turf field leachate containing CR materials included 15 PAHs, of which 5 PAHs (i.e., ANT, BaA, BghiP, BkF, and DahA) had alarming concentrations. In another study, Celeiro et al.^66^ found that the leachate samples collected from several artificial turf football pitches containing CR as infill may include approximately 16 different PAHs, of which 8 PAHs (i.e., ACE, ANC, CHY, FLA, FLU, NAP, PHN, and PYR) had alarming concentrations. Llompart et al.^67^ assessed the process of evaporating samples derived from commercial pavements and playgrounds manufactured from recycled rubber at 25°C and 60°C. During the experiment, some pavement samples contained benzo[*a*]pyrene (BaP)—the most carcinogenic PAHs—as well as all other carcinogenic compounds, except for CHY and B[*a*]A. This reflects the daily exposure of athletes to chronic toxicity from PAHs derived from recycled tires utilized on playgrounds.^52^

The need for increasingly controlling the quality of granules regarding hazardous substances is being affected by the EU requirements for PAH content in rubber granules utilized in sports fields. If new permissible limits are apparently disregarded, alternative directions must be increasingly sought for their application.^53^

### Heavy metals

Heavy metals (e.g., Pb and Zn) are incorporated into the tire product manufacturing process. While Zn generally serves as an activator, Pb is commonly used as a pigment and heat stabilizer.^68^ Zn is a toxic metal with a highest concentration than other toxic metals.^34^ ZnO is employed as an activator in tire vulcanization to enhance the efficiency of the curing stage across the world and strongly contributes to the properties of tires, including rolling resistance.^69^ It also accounts for a mass fraction of 1–2% in tire rubber.^34^ The Zn content of tire wear plays a key role in its impacts on human toxicity (72%) and marine toxicity (71%). It also accounts for 40% of the vehicle impact on terrestrial toxicity.^69^ In general, heavy metals are non-biodegradable. As a result, after being taken into animal bodies, they may bioaccumulate in organisms. When present in large amounts, a number of heavy metals cause toxicity, leading to physiological and biological complications.^34^ Humans are exposed to CR toxins potentially via ingestion, contact, or inhalation, depending on the particular purpose of playing surfaces and environmental factors such as temperature. For instance, heavy metals may be inadvertently ingested from particles,^2^ which accumulate in the body and adversely impact organs and systems, including the nervous system and the hematopoietic system.^53^ Pb and Cd exposure leads to increasing earthworm mortality and decreasing fertility and body weight. Besides, Zn ions in solution are extremely toxic to invertebrates, vertebrate fish, and plants.^64^

According to reviewed studies, the environmental compartments mediate the release of different heavy metals, especially Cd and Pb, and fairly large quantities of Zn, from diverse scrap tire applications. Once discharged into the environment, these heavy metals adversely affect the environment itself and organisms. The activity of both earthworms and microorganisms can be altered when these compounds are leached into the turf field due to diverse organic compounds, the existence of heavy metals in substantial quantities, and the presence of hydrocarbons in CRs.^51^ Pochron et al.^64^ assessed the impact of the utilized CRs derived from recycled tires in turf fields on indigenous earthworms and bacteria. The lifespan, activity, and survival of both indigenous earthworms and microorganisms (under light and heat stresses) were examined. The results suggested that CR contamination in soil did not affect the microbial respiration rate. Conversely, the body weight of earthworms living in contaminated soils was lower than that of those living in uncontaminated soils. Except for Zn, the soil concentration of other reviewed heavy metals (e.g., As, Pb, Cd, Cu, Ni, and Hg) did not surpass the permissible limit announced. Shalaby et al.^70^ sought to shed light on the potential effects of the use of scrap tires in full-scale construction of road embankments by collecting and examining multiple representative samples. According to the findings, Al, Mn, and Fe concentrations in the water samples derived from the underground water source situated under embankments exceeded the established levels. However, there was no need to urgently address these heavy metals as they were secondary potable water quality parameters.

Lu et al.^65^ demonstrated that, after UV exposure, tire CRs utilized in artificial turf fields can release 0.2 to 1.3 μg/g of Zn into leachates. Also, Azizian et al.^71^ evaluated the concentrations of different heavy metals in leachates near the roads under construction with tire rubbers. The analysis results showed that while Al, Ca, Hg, K, Mg, P, and Sr concentrations significantly exceeded the analyzer detection limit, those of the remaining thirteen heavy metals (i.e., As, Ba, Cd, Cr, Co, Cu, Fe, Ni, Pb, Se, Sb, V, and Zn) fell below it. Kalbe et al.^72^ argued that the use of scrap tires as granular materials in sports field construction may release different organic and inorganic toxins into the groundwater and soil. Laboratory experiments indicated that aging utilized tire scraps by artificial weathering gives rise to leachates containing high Zn levels.

### VOCs and airborne pollution

The problem of VOC emissions from ground tire rubbers, particularly those used in artificial turf fields, has recently garnered increasing attention, as characterized by independent research teams.^73^ VOC exposure has been found to increase earthworm mortality.^64^ Tires directly release numerous VOCs and semi-VOCs (SVOCs) into the air. Efforts have been made to detect common VOCs migrating from rubber. The association of benzene, toluene, xylene (BTEX), their derivatives, and other VOCs with tire CR has been explored.^74^ This is because during degradation or devulcanization/reclamation of rubber, emissions of low-molecular-weight VOCs connected with disintegrating cross-linked bonds, main chain scission, and evaporation of rubber additives such as plasticizers naturally occur.^73^

Gagol et al.^75^ confirmed a strong correlation between VOC emission efficiency and GTR devulcanization degree. A co-rotating twin screw extruder was used to thermomechanically treat GTR at various temperatures (80, 120, and 160°C). The maximum VOC concentration of GTR was obtained at 160°C. According to the results, emitted VOCs interacted with one another during extrusion to form new chemical structures, thereby affecting the total amount of VOCs.

Ruffino et al.^54^ concluded that benzene, BTEX, and toluene could be released into leachates as a result of using artificial turf. Moreover, BTEX was detected in leachates of new infill materials in higher concentrations than its older counterparts. As reported by Azizian et al.,^71^ several compounds were found in collected leachate samples released from CR materials utilized as construction materials on highways. These included pyrido, 10-(benzyloxy)-6,7-dihydro-,tetramethyl ester, benzothiazole, phthalic anhydride, silver(1+) salt, benzaldehyde, 3-hydroxy-4-methoxy, glycine, N-methyl-N-(1-oxododecyl), hexadecanoic acid, quinoline, 2,3-dimethyl,[1,1-Biphenyl]-2-ol2(3H)-benzothiazolone, and decanoic acid. Based on the results, benzothiazole could be removed from the generated leachates through various environmental processes, e.g., soil absorption, biodegradation, and volatilization.

Tandon et al.^76^ reviewed representative air samples gathered from a given site where tire chips were used as infill materials for embankments. The samples consisted of various VOCs, including 1,4-dichlorobenzene, ethylbenzene, dichlorodifluoromethane, tetrachloroethylene, styrene, trichloroethylene, toluene, trichlorofluoromethane, 1,3,5-trimethylbenzene, 1,2,4-trimethylbenzene, o-xylene, and M&p-Xylene. Also, Schneider et al.^45^ witnessed different VOCs (e.g., tert-butylamine, 2-heptanone, cyclohexane, benzothiazole, and saturated aliphatic hydrocarbons NC9) contained in certain samples present at sites using rubber granules as infill materials for artificial turf.

Reactive extrusion is among the most promising green solvent-free continuous processes with diminished heat demand that exploit commonly available equipment in polymer technology.^77^ It also allows for the application of additional GTR modifiers. Nonetheless, rising temperatures and considerable shear forces acting on the material during thermomechanical treatment might cause GTR to partially decompose. This has several drawbacks despite the aforementioned advantages, including VOC emissions during modification and from the final product (i.e., devulcanized GTR) as the most serious one.^75^ These VOC emissions can be due to evaporated additives (curing agents, plasticizers, or their degradation products) applied throughout rubber manufacturing. Furthermore, volatile aromatic/aliphatic hydrocarbons, ketones, and aldehydes may form due to the decomposed macromolecular chains of synthetic and natural rubbers, often posing environmental and human health risks.^78^

In light of the foregoing, a key GTR management issue is the emission of VOCs, including harmful compounds to the environment and the human body. This aspect is linked to both GTR chemical composition and their physical properties (e.g., surface development or particle size) and can be affected by different treatments intended for devising GTR management strategies.^79^

### Sulfur emissions in tire recycling and reuse processes

Sulfur has been used in tire manufacturing to vulcanize styrene butadiene; sulfur content of a typical tire is around 1.6 wt %.^80^ Many of recycling methods liberate sulfur in various forms. For instance, the pyrolysis process, which takes place in the absence of air at temperatures varying from 400°C to 700°C leads to thermal degradation of sulfide bonds in the rubber framework, producing liquefied rubber, as well as gaseous and solid by-products. This results in the release of hydrogen sulfide (H_2_S), carbonyl sulfide (COS), sulfur dioxide (SO_2_), and other sulphurous gases.^80^^,^^81^ Hydrogen sulfide (H_2_S) can be oxidized to sulfur di-oxide (SO_2_) or sulfur trioxide (SO_3_) and finally form sulfates.^80^^,^^81^ It has been documented that in the pyrolysis of scrap tires, nearly half of the total content of sulfur remains in the solid by-product, mostly in the form of ZnS and aliphatic sulfide.^82^^,^^83^ The liquefied rubber contains thiophene, benzo-thiophene and di-benzo-thiophene.^83^

Compared to pyrolysis, incineration of tire emits sulfur oxides (SO_x_) resulting from combustion compounds containing sulfur in rubber.^84^ It was found out that the sulfur di-oxide (SO_2_) emissions were amplified as the temperature increased and peaked at 750°C.^85^ Sulfur could also leach from GTR, which is used in playgrounds as well as construction of concrete and asphalt roads and parking lots.^78^ Jeong et al.^86^ explored the trends of chemical leaching from tires centered on the treadwear rating of the tire. Liquid chromatography–tandem mass spectrometry was used to analyze the chemical leaching, with a particular focus on benzothiazole and its derivative 2-hydroxy benzothiazole. However, chemical mapping via high-resolution tandem mass spectrometry detected another derivative: 2-mercaptobenzothiazole. Selbes et al.^87^ showed that sulfur was present in leachates from not only crumb rubber but also in tire chips. Sulfur leached quicker in the early days of exposure but then gradually slowed down over time. This revealed that the sulfur compounds can mobilize in aqueous conditions. Similarly, Duda et al.^88^ demonstrated that sulfate concentration rose from 0 to 273 mg/L when tire bales were submerged for 120 days. Hennebert et al.^89^ reported sulfur levels of 217 mg/kg in leaching, 19.24 mg/kg in lysimeter tests, and 313 mg/kg in percolation from shredded tire rubber.

### 6PPD and 6PPD-Q: Contaminants of emerging concern

6PPD (*N*-(1,3-dimethyl butyl)-*N*″-phenyl-*p*-phenylenediamine) is an antioxidant widely used to delay thermo-oxidative tire aging. A thin 6PPD layer on tires performs antiozonant activity as it provides protection from oxygen and atmospheric ozone. Therefore, a variety of transformation products are generated.^90^ The ozone protection mechanism of 6PPD arises from the reactions in which *p*-phenylenediamine moieties donate electrons to the O_3_ molecules.^91^ 6PPD is among the most widely used antioxidants and has been classified as a high production volume chemical. China produced 200,000 tons of 6PPD in 2020, representing 54% of the total rubbery antioxidant production.^92^ 6PPD has been estimated to be present between 0.4% and 2% (by rubber mass) in all commercial and passenger vehicle tires.^93^ Approximately 1–2% of the 6PPD content in tires gradually moves to the surface during tire operation, leading to brown discoloration and a decrease in 6PPD concentration over time.^91^ It may also be released into the surrounding environment during the rubber manufacturing and utilized rubber article recycling processes.^94^ In the environment, 6PPD oxidizes into its byproduct, i.e., 6PPD-quinone (6PPDQ)^95^ when exposed to ozone. This ozonation process is a three-step chain reaction: (1) formation of a 6PPD amine oxide (C_18_H_24_NO^−^) due to oxygen (O_2_) loss, (2) formation of the side chain, and (3) final dissociation to 6PPDQ (C_18_H_22_N_2_O_2_).^94^ Nevertheless, 6PPDQ is a 6PPD derivative with greater environmental persistence and stability, and it was found in larger quantities than 6PPD in the river.^96^

Researchers began studying this subject a few decades ago (as documented by Scholz et al.^97^). However, clearer insights have recently been offered into how 6PPD-linked compounds are widely released into the environment as well as how they are conspicuously toxic to aquatic organisms. Particularly, in 2021, 6PPD-q was found to be a causative toxicant for the significant mortality of coho salmon in Seattle, USA.^98^ 6PPDQ is toxic not only to coho salmon but also to other fish species, including brook trout, rainbow trout, chinook salmon, steelhead, white-spotted char, and zebrafish larvae.^99^ In this respect, 6PPDQ leaks from tire dust can reach an equilibrium concentration in adjacent aquatic media in a short time. This appears to be the explanation for increased 6PPDQ content in runoff. The hydrolysis-based degradation of 6PPDQ has been proposed, with the half-life estimated to be 13–16 days^100^ Several studies have recently reported their toxic potency to organisms like fish, mussels, crustacean species, and vertebrate embryos. In recent years, increasing attention has been devoted to their prevalence in the environment as well as their potential negative effects.^101^ The California State Assembly enacted and accepted the S.A.L.M.O.N Act (Saving Aquatic Life from Manufactured Oxidized Nano-chemicals) in March 2024. Caltrans receives direction from this legislation to create and enforce methods that stop 6PPD from entering salmon- and trout-bearing waters by relying on biofiltration and bioretention systems. Multiple scientific studies spanning decades have confirmed that 6PPDQ causes huge salmon mortality during storms, thus propelling this environmental policy into alignment with scientific findings.^102^

A high risk of human exposure to 6PPD and 6PPDQ, apart from their ecological risk, has been reported.^103^ These compounds have been detected in CR, atmospheric particles, air, consumer products, playground dust, and indoor dust.^103^ They have also been found in wastewater, runoff, and surface water,^99^ and soil.^101^ As they have anthropogenic sources, including atmospheric particles, indoor dust, and runoff, humans may be exposed to 6PPD and 6PPDQ through skin contact, inhalation, or diet.^99^ Fine particle inhalation, contaminated water, and dietary intake may lead to human exposure to 6PPD and 6PPDQ.^104^ They have been found in human urine, with the highest exposure to these chemicals observed in pregnant women, followed by non-pregnant adults and then children.^103^ Moreover, Ji et al.^105^ were the first to find 6PPD and 6PPD-q in fish samples, highlighting their potential human health risks through ingesting contaminated fish. Besides, Du et al.^92^ have already reported their presence in human urine. This indicates the urgent need to consider potential risks to human health caused by their exposure. Song et al.^106^ demonstrated that liver damage can be linked with 6PPD and 6PPDQ exposure.

Potential downstream metabolites of rubber-derived quinone compounds, including 6PPDQ, are yet to be evaluated for mammals, despite the literature on PPDQ biotransformation pathways and reports on the resulting biotoxicity. Researchers have been increasingly investigating 6PPD and 6PPDQ aquatic toxicity; however, their impacts on mammals remain insufficiently documented.^99^ There is a small body of research on the biotransformation pathways of 6PPDQ, and earlier studies primarily providing *in vitro* models or evaluated aquatic organisms. Hence, further research is needed, particularly using *in vivo* models of humans and rodents, to better understand its effects.^107^^,^^108^^,^^109^ It is necessary to investigate the biotransformation pathways of 6PPDQ in humans and other mammals to characterize its biological fate in these organisms. This will help identify species-specific differences in 6PPDQ metabolism and assess its potential adverse impacts on human health and ecosystems.^99^

Prompted by recent findings, the international community expressed concerns over 6PPD and its derivative 6PPDQ. This highlighted the pressing necessity for conducting additional research and formulating environmental management strategies to alleviate the detrimental effects of these compounds. It is crucial to monitor the presence of 6PPDQ since it is unsurprisingly widespread globally in road-affected settings and has been identified in dust, fine particles, and surface water. Very little is known about the pitfalls of exposure and toxicity to humans and other organisms. Nevertheless, since 6PPDQ is ubiquitous and has potential toxicological impacts, it is vital to understand its environmental fate and implement proper management strategies.^98^

6PPDQ can also be found in urban air particulate matter containing tire dust and indoor dust, particularly those from rubber recycling plants.^100^ As an exemplary rubber recycling application, rubber granules are used as infill for artificial turf. Nonetheless, these granules may inevitably be dispersed into the environment since they are loosely applied to the surface. In the absence of abatement measures, it is estimated that ∼950 kg of rubber particles would leave lawns each year. Recent studies have indicated high 6PPD levels of uncoated rubber infill materials. Interestingly, research revealed lower 6PPD concentrations (e.g., 65.67 and 119.20 mg/kg) of colored polyurethane-coated rubber infill, originating from both recycling company collections and sports field turf, in comparison with uncoated particles (e.g., 570.53 and 1,478.60 mg/kg). This implies that 6PPD release may be influenced by the surface contact area. Furthermore, a comparison of recycled rubber granules indicated significantly higher 6PPD concentrations in granules directly collected from recycling companies compared to those obtained from sports fields. However, since the specific service age was not determined for the rubber samples, whether sunlight exposure-mediated 6PPD degradation or rubber sample freshness variations have led to the observed disparities remains uncertain. Besides advancing efforts devoted to rubber waste recycling, the potential for replacing 6PPD with other PPDs has been explored by some researchers by evaluating the structural properties of compounds in the 6PPD family. However, there is still scant research in the recent literature on the identification of ideal substitutes for 6PPD as a rubber antioxidant.^98^

It is essential to evaluate 6PPD/6PPDQ derived from recycled tire rubber (RTR)-modified asphalt to ensure the sustainable reuse of RTR in pavement design. The physical properties of asphalt, such as surface area and air voids, along with its chemical composition and the chemistry of stormwater, are major factors influencing the complex release mechanism of 6PPDQ from rubberized asphalt mixture.^110^ Additionally, 6PPDQ released from tires primarily interacts directly with asphalt pavement,^111^ yet its behavior at the asphalt concrete (AC)–water interface remains a significant knowledge gap in understanding its environmental dynamics. However, some studies have investigated metal sorption onto porous asphalt concrete pavements or asphalt mixtures, but research on the sorption of organic pollutants by such mixtures is still limited.^112^ Introducing RTR into asphalt mixtures may affect the sorption of organic compounds due to the addition of organic carbon to the asphalt binder. As a relatively hydrophobic compound with low water solubility, 6PPDQ requires further investigation into its sorption and desorption mechanisms at the AC–water interface.^110^

**Figure 2:**
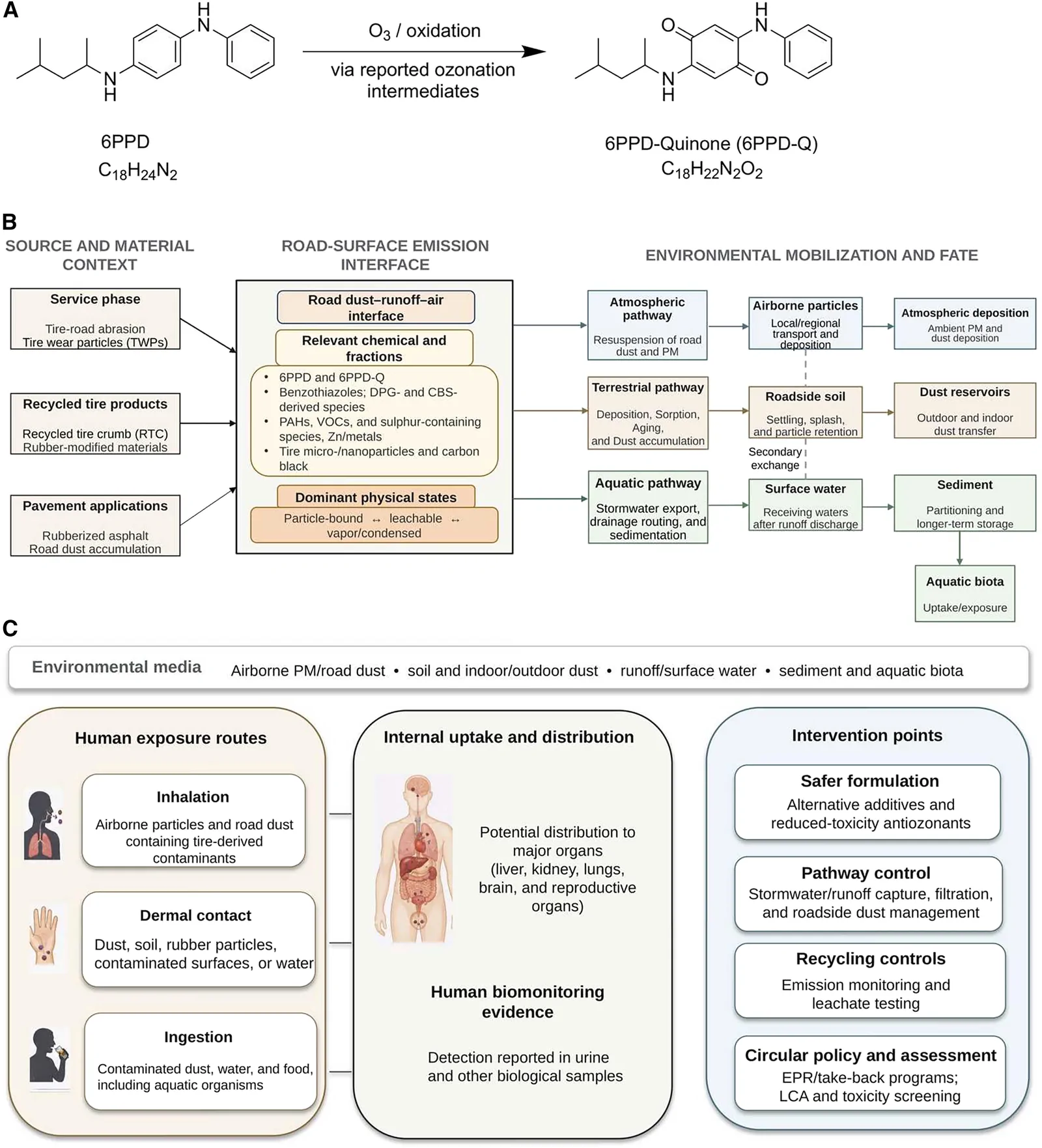
Sources, transformation, environmental fate, and exposure pathways of tire-derived 6PPD and 6PPD-Q (A) Atmospheric oxidation of 6PPD by ozone yields 6PPD-quinone through multiple reported reaction intermediates. (B) Environmental mobilization through air, soil, runoff, water, sediment, and biota. (C) Human exposure, uptake, and intervention points. Abbreviations: DPG (1,3-diphenylguanidine); CBS (N-cyclohexyl-2-benzothiazolesulfenamide); EPR (Extended Producer Responsibility); LCA, (Life Cycle Assessment).

To provide a comprehensive illustration of the sources of 6PPD and 6PPDQ generation, the pathways through which they contaminate the environment, as well as their effects on human health, Figure 2 has been presented. To summarize, researchers continue to investigate the long-term health effects of bioaccumulated 6PPD and its derivatives. Given its lethal impact on aquatic ecosystems, 6PPD is also expected to pose risks to human health. Consequently, effective techniques should be developed for the isolation and removal of these harmful compounds from discarded CR. In addition to designing procedures for isolating specific additives, the relatively low concentration of 6PPD in CR (approximately 2 wt %) presents a significant challenge, further complicating the separation process. While there is strong momentum toward replacing 6PPD in tire manufacturing, it remains essential to manage the existing 6PPD content in CR and to prevent its release from landfilled waste into the environment.^113^

### Tire microplastics (TMPs)

Microplastics (MPs) are a widespread global pollutant present across ecosystems and capable of affecting terrestrial and aquatic organisms, including humans, through exposure pathways such as air, water, and the food chain. Among them, TMPs derived from synthetic rubber tires are major contributors to environmental contamination. This use-phase source is especially important because tire wear particles are generated continuously during normal driving, meaning that tire-derived microplastic pollution is not limited to recycling, landfilling, or other end-of-life pathways.^2^^,^^12^^,^^14^ TMPs primarily include tire wear particles (TWPs), generated by friction between tires and road surfaces; recycled tire crumb (RTC), produced from shredded tires for applications such as artificial turf and recreational surfaces; and tire repair-polished debris (TRD), formed during tire repair through mechanical polishing of the inner liner.

Similar to conventional plastics, TMPs persist in the environment and progressively accumulate across environmental matrices. They are considered the second-largest primary source of marine microplastic pollution. Although recent studies have increasingly focused on TWPs, limited attention has been given to other TMP sources, such as TRD and RTC. In particular, despite concerns regarding the health and safety impacts of RTC used in artificial turf and playgrounds, its environmental behavior and ecological risks remain insufficiently understood.

Previous publications^114^^,^^115^^,^^116^ have often neglected RTC as a TMP source, whereas its emissions should be considered due to its widespread application. Given various TMP generation methods, TWPs, TRD, and RTC may differ in terms of potential impacts and environmental fates. The relationships between various types of TMPs have not yet been uncovered due to their poorly documented comparison. Thus, further research is needed on TMPs to gain deeper insights into the environmental fates, occurrences, impacts, behaviors, and potential solutions of TMPs, especially TRD and RTC.^8^

### Nano carbon black

Car tires contain fillers such as carbon black (CB), ZnO, clay, and silica, with rubber and CB comprising approximately 45 wt % and 30 wt % of tire composition, respectively. In TWPs, CB is embedded within the rubber matrix but can become exposed through abrasion and weathering, leading to the release of both micrometer-sized TWPs and nanoscale CB particles into the environment. Consequently, CB may contribute to particulate matter formation and nanoparticle exposure. Fine micro- and nanoparticles are commonly generated from bulk materials through top-down grinding methods such as ball milling.

Although CB is generally considered tightly bound within the tire rubber matrix and unlikely to be released during tire wear, environmental weathering processes such as temperature fluctuations, road abrasion, and dust resuspension can fragment TWPs. This fragmentation may subsequently release free-bound CB particles into environmental media, potentially contributing to particulate and nanoparticle exposure.^117^

### Life cycle assessments of tire recycling

In addition to the available regulations and standards, understanding the sustainability performance of the tire industry requires quantitatively analyzing the environmental impacts of tires throughout their life cycle. LCA is an efficient quantitative approach to evaluate the environmental impacts of a product or a process from cradle-to-grave.^9^ Therefore, it has been employed during recent decades as an essential tool to assess the environmental impacts of different products and cycles of chemical processes. It also proposes a methodology for the assessment and analysis of the pros and cons of waste management strategies and systems, including incineration, landfill, and chemical/mechanical recycling from an environmental viewpoint. Different countries have implemented LCA programs for waste [type] management, decision-making, and strategic planning.^22^ LCA was first applied in the tire industry in the 1990s to compare filler types and evaluate the environmental impacts of tires, among others. Since the 2010s, researchers have become increasingly focused on comparative LCA studies on ELTs by adopting new treatment approaches. Although LCA on tires has been discussed in a dozen studies within the past two decades, LCA applications in the industry may be challenged by inconsistent methodology and misinterpreted sustainability results.^9^

LCA analysis has also been evaluated in certain reviewed studies. For instance, Fiksel et al.^118^ aimed to identify a green alternative to scrap tires using the LCA approach. Based on their findings, tire recycling for artificial turf and cement purposes could lead to diminished air toxics, GHG emissions, and water supply use. Furthermore, almost 543 kg of both direct and indirect GHG emissions in terms of CO_2_ equivalent (CO_2_-eq) would be avoided per metric ton of tire-derived fuel (TDF) used as an alternative in cement kilns. Magnusson et al.^119^ evaluated the environmental impacts of recycled tires used as infill for artificial turf. They concluded that using recycled tires can lead to minimized GHG emissions and energy consumption. Besides, the obtained samples depicted measurable Zn concentrations in the recycled tire leachate. Piao et al.^120^ investigated the potential environmental implications of utilizing CR materials in rubberized semi-dense asphalt (SDA) pavements by adopting the LCA approach. According to the findings, CR products should not be used since PAHs may enter the groundwater.

Additionally, Neri et al.^121^ compared the pyrolysis of used tires with other valorization techniques by applying an LCA methodology. The selected unit was 1 ton of ELTs treated. Different scenarios were investigated using the ReCiPe impact assessment method. Tire pyrolysis caused lower environmental impacts than incineration and other energy recovery processes. It also involved the recovery of secondary materials (CB, steel, and oil) more efficiently and with lower cumulative energy (130 MJ_eq_⋅ton^−1^) compared to conventional crushing (1830 MJeq&middotton^−1^). However, while pyrolysis poses more environmental problems, treated pyrolysis oil mitigates environmental problems. Generally, given the very limited use of GTR and the much greater applicability of pyrolysis once generalized, tire pyrolysis could dramatically reduce environmental burdens, i.e., ELTs.

The range of material recovery carbon emissions is -3217–667 kg CO_2_-eq per ton of tires, with the former due to the rubber reused for synthetic turf, avoiding raw materials production, and the latter caused by grinding during asphalt manufacturing. Also, the range of energy recovery carbon emissions is −1466 to −500 kg CO_2_ eq. Carbon emissions are reduced on average by 748 and 901 kg CO_2_-eq for energy and material recovery, respectively, suggesting the superiority of material recovery over energy recovery for carbon emissions reduction.^9^

Generally, most LCA studies assume that more comprehensive ELT recycling, allowing for the recovery of an increasing number of individual tire components (e.g., CB, steel, textiles, and rubber), will lead to a minimal carbon footprint. Moreover, the environmental impacts of tire recycling can be mitigated by using electricity produced by green energy. Overall, in comparison with landfilling and incineration, tire retreading, using degraded GTR in tire remanufacturing, dissolution methods, and separation of different components can substantially alleviate environmental problems. Meanwhile, since tires are inadequately applied in recycling industries, such as dissolution methods, tire remanufacturing, and polymer-modified bitumen, the pyrolysis process, which can result in frequently used products, is regarded as the most reliable tire valorization method.^22^

### Circular-by-design tire recycling technologies

The growing body of evidence regarding the environmental and human health risks associated with scrap tire recycling highlights the urgent need to redesign future recycling systems according to circular economy principles. While current approaches such as dynamic desulphurization, pyrolysis, combustion, and chemical devulcanization enable partial recovery of material or energy value, many remain energy-intensive and generate secondary pollutants. Consequently, next-generation tire recycling technologies should move beyond simple waste management strategies toward “circular-by-design” systems that integrate material safety, low-carbon processing, and closed-loop recovery from the earliest stages of material and process development.

**Figure 3:**
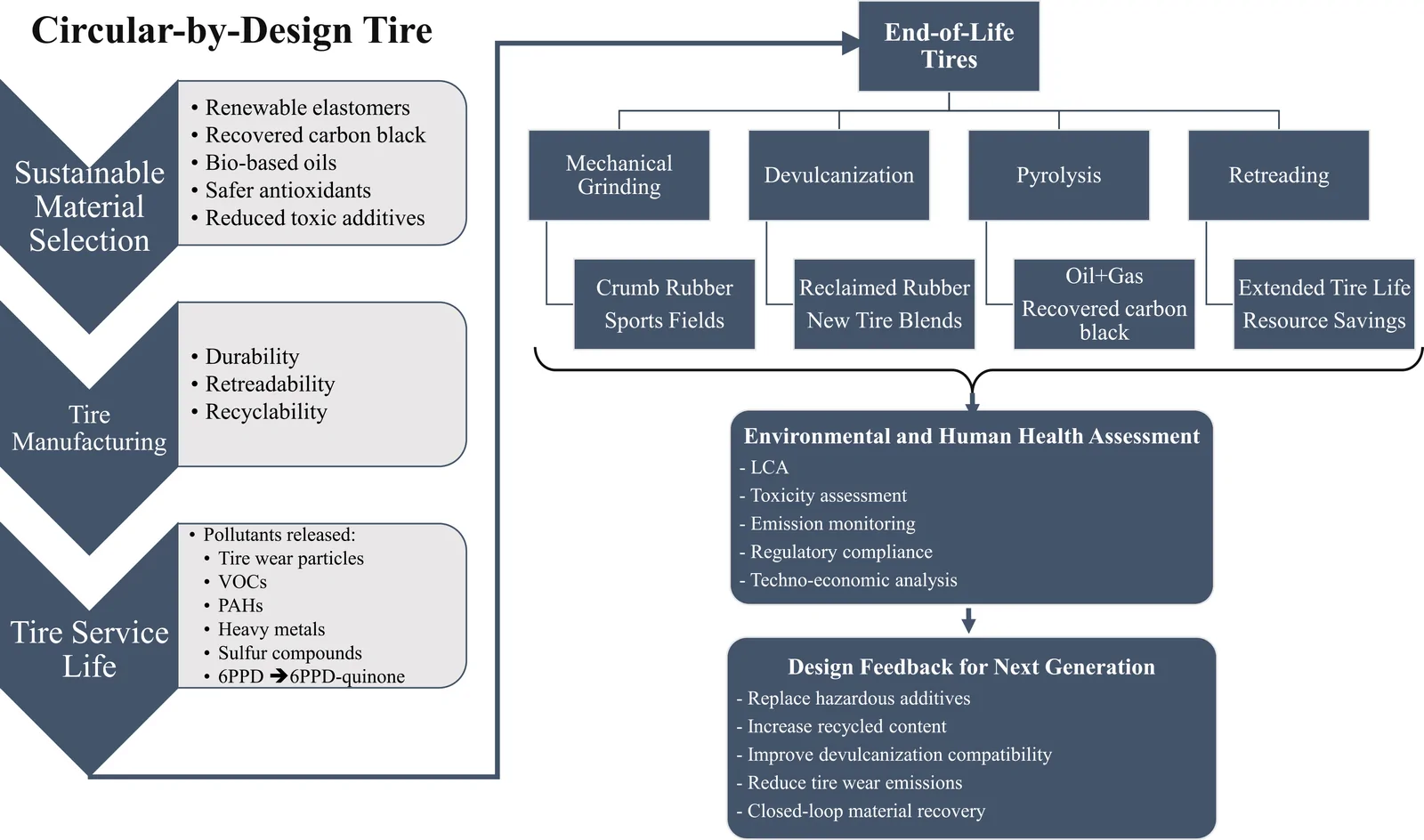
Circular-by-design framework linking tire material selection and manufacture with service-life emissions, end-of-life recovery pathways, sustainability assessment, and design feedback for the next generation of tires

To translate these principles into an integrated decision framework, Figure 3 presents a circular-by-design approach covering the entire tire life cycle. The framework begins with safer and more sustainable material selection, extends through manufacturing and the service phase, and connects these upstream decisions with the principal end-of-life pathways, including mechanical grinding, devulcanization, pyrolysis, and retreading. Importantly, these pathways are not assumed to be sustainable solely because they divert tires from disposal. Instead, their recovered products, energy requirements, emissions, and potential human health effects must be evaluated using LCA, toxicity assessment, emission monitoring, regulatory compliance, and techno-economic analysis. The findings from these assessments are subsequently fed back into the design of the next generation of tires, creating an iterative pathway toward safer additives, greater recycled content, lower wear emissions, improved devulcanization compatibility, and closed-loop material recovery.

As illustrated in Figure 3, circularity should begin before a tire reaches the end-of-life stage. One promising upstream intervention involves developing tire formulations specifically engineered for recyclability. Conventional tires are based on highly crosslinked sulfur-cured elastomers, which complicate selective devulcanization and material recovery. Recent studies have therefore proposed dynamic covalent networks, reversible crosslinking chemistries, and vitrimer-based elastomers that retain mechanical durability during use while enabling controlled depolymerization or reprocessing at the end of life.^122^ Incorporating reversible sulfur bonds or exchangeable crosslinking motifs into tire compounds could substantially reduce the energy demand of recycling while preserving polymer chain integrity.^123^

Another critical strategy is the replacement of hazardous additives and processing chemicals with greener alternatives. Conventional reclaiming and devulcanization often rely on aromatic oils, toxic softeners, and odorous chemical activators that contribute to environmental emissions and occupational exposure risks.^124^ Future systems should prioritize bio-based plasticizers, low-toxicity solvents, ionic liquids, supercritical fluids, and catalyst-assisted selective sulfur cleavage processes that minimize harmful byproducts.^48^^,^^125^ In particular, microwave-assisted and mechanochemical devulcanization technologies have attracted attention because they can selectively break sulfur crosslinks with lower chemical consumption and shorter processing times compared with conventional thermo-chemical methods.^126^

Decarbonization and energy efficiency must also become central criteria in future recycling infrastructure. Pyrolysis and thermal recovery remain commercially attractive because of their scalability and capacity to recover energy-rich products; however, they are often associated with substantial carbon emissions and complex gas-cleaning requirements.^127^ Integrating renewable electricity, electrified reactors, heat recovery systems, and carbon capture technologies into thermal recycling facilities could significantly reduce life cycle emissions. Furthermore, coupling pyrolysis with high-value carbon black upgrading rather than fuel production may improve both circularity and economic viability by preserving material value within the manufacturing cycle.^128^

The unintended environmental consequences identified in recent years also emphasize the importance of comprehensive life cycle thinking. Research on tire wear particles and microplastics has demonstrated that recycling cannot be considered sustainable solely because waste is diverted from landfills; rather, the full environmental fate of recycled products must be evaluated.^12^ Future recycling technologies should therefore integrate life cycle assessment (LCA), toxicological screening, and safe-and-sustainable-by-design (SSbD) frameworks to identify environmental trade-offs during process development rather than after commercialization. Such approaches are increasingly recognized within European sustainability policy and advanced materials research initiatives.^129^

Digitalization and advanced material monitoring may further support circular tire systems. Emerging technologies such as AI-assisted sorting, digital product passports, tracer-based material identification, and real-time emission monitoring could improve the quality and traceability of recovered rubber streams while facilitating regulatory compliance and secondary material certification.^130^ These tools may help create closed-loop recycling ecosystems in which recovered tire materials can be continuously reintegrated into high-performance applications rather than downcycled into low-value products.

Ultimately, the future of tire recycling will likely depend on integrating material innovation, green chemistry, process electrification, life cycle assessment, and environmental health considerations into a unified circular framework. The unintended consequences associated with current recycling practices should not only be viewed as limitations, but also as critical drivers for innovation. By embedding circularity, safety, and sustainability directly into tire design and recycling technologies, the industry can transition from waste mitigation toward truly regenerative material systems.

## Discussion

Recycling and many other sustainability interventions are often framed as inherently beneficial, yet they are constrained by an “impossible triangle” of environmental protection, economic viability, and resource efficiency. In practice, improving one dimension frequently creates trade-offs in the others: increasing material recovery or performance can require energy-intensive processes, additional chemical inputs, or complex infrastructure that raise emissions and costs, while prioritizing low cost or minimal environmental controls can reduce output quality, limit recovery rates, or increase contamination risks. As a result, environmental burdens are often not eliminated but shifted between air, water, and soil compartments, raising concerns that some solutions may represent burden shifting or even greenwashing rather than true sustainability gains. Genuine progress therefore requires a systems-level assessment.

The present review is an attempt to determine if recycling scrap tires can serve as a sustainable solution or result in potential human health and environmental risks. In accordance with the obtained evidence, even though recycling presents landfill reduction, resource conservation, and partial compliance with circular economy targets, it may result in underappreciated and serious challenges as well. Although mechanical recycling of rubber into ground tire rubber (GTR) enables its reuse in applications such as construction materials, asphalt modification, polymer composites, backfilling, and turf systems, it also raises concerns regarding the potential leaching of hazardous substances and the generation of microplastic pollution. Even though devulcanization is technically encouraging, it is still limited by the release of secondary emissions, scalability, and cost. While pyrolysis presents material recovery pathways, it remains constrained by economic competitiveness and the air pollution hazards if emissions are left loosely controlled. Of all pathways, toxic emissions-such as VOCs, PAHs, sulfur compounds, heavy metals, 6PPD/6PPD-quinone, and tire microplastics-may impose human health and ecological risks. As persistent contaminants capable of polluting air, water, and soil, these substances raise important concerns about the long-term safety of recycled products. Without adequately accounting for potential human health and environmental impacts, claims of sustainability remain incomplete. While most existing review studies primarily focus on technical performance and economic considerations, the present paper advances the discussion by integrating regulatory developments, environmental toxicology, and circular economy perspectives to provide a more comprehensive and holistic assessment of sustainability. The conclusion is nuanced, as “one may not consider the present recycling solutions as completely sustainable solutions.” Although these strategies present partial advantages, they are still constrained by inconsistent economic viability, insufficient regulatory oversight, and pollutant release. The innovative aspect of the present review lies in the explicit identification of such discrepancies and framing tire recycling as a dual-edged practice-mitigating waste on one hand, but generating new risks on the other.

## Acknowledgments

The authors did not receive any funding for this study.

## Author contributions

Conceptualization, E.H.F.; data curation, M.V., P.H., and S.R.; visualization, M.V., P.H., and S.R.; writing – original draft, M.V.; writing – review and editing, M.V., P.H., S.R., and E.H.F.; supervision, E.H.F.

## Declaration of interests

The authors declare no competing financial or non-financial interests.

## Declaration of generative AI and AI-assisted technologies in the writing process

During the preparation of this work, the authors used ChatGPT in order to produce Figures 1 and 2 based on the concept and design specifications developed by the authors. After using this tool, the authors reviewed and edited the content as needed and take full responsibility for the content of the publication. These figures are intended solely to enhance communication and improve the clarity of the concepts and summaries discussed, and do not contain or represent technical data.

## References

[1] Vahdatbin M., Hajikarimi P., Fini E.H. (2025). Devulcanization of Waste Tire Rubber via Microwave and Biological Methods: A Review. Polymers.

[2] Mayer P.M., Moran K.D., Miller E.L., Brander S.M., Harper S., Garcia-Jaramillo M., Carrasco-Navarro V., Ho K.T., Burgess R.M., Thornton Hampton L.M. (2024). Where the Rubber Meets the Road: Emerging Environmental Impacts of Tire Wear Particles and Their Chemical Cocktails. Sci. Total Environ..

[3] Maroufi S., Khayyam Nekouei R., Sahajwalla V. (2017). Thermal Isolation of Rare Earth Oxides from Nd–Fe–B Magnets Using Carbon from Waste Tyres. ACS Sustain. Chem. Eng..

[4] Zhi M., Yang F., Meng F., Li M., Manivannan A., Wu N. (2014). Effects of Pore Structure on Performance of An Activated-Carbon Supercapacitor Electrode Recycled from Scrap Waste Tires. ACS Sustain. Chem. Eng..

[5] Wang S., Cheng M., Xie M., Yang Y., Liu T., Zhou T., Cen Q., Liu Z., Li B. (2025). From Waste to Energy: Comprehensive Understanding of the Thermal-Chemical Utilization Techniques for Waste Tire Recycling. Renew. Sustain. Energy Rev..

[6] Ferdous W., Manalo A., Siddique R., Mendis P., Zhuge Y., Wong H.S., Lokuge W., Aravinthan T., Schubel P. (2021). Recycling of Landfill Wastes (Tyres, Plastics and Glass) in Construction – A Review on Global Waste Generation, Performance, Application and Future Opportunities. Resour. Conserv. Recycl..

[7] Fohet L., Andanson J.-M., Charbouillot T., Malosse L., Leremboure M., Delor-Jestin F., Verney V. (2023). Time-Concentration Profiles of Tire Particle Additives and Transformation Products under Natural and Artificial Aging. Sci. Total Environ..

[8] Luo Z., Zhou X., Su Y., Wang H., Yu R., Zhou S., Xu E.G., Xing B. (2021). Environmental Occurrence, Fate, Impact, and Potential Solution of Tire Microplastics: Similarities and Differences with Tire Wear Particles. Sci. Total Environ..

[9] Dong Y., Zhao Y., Hossain M.U., He Y., Liu P. (2021). Life Cycle Assessment of Vehicle Tires: A Systematic Review. Clean. Environ. Syst..

[10] Hennequin T., Huijbregts M.A.J., van Zelm R. (2023). The Influence of Consumer Behavior on the Environmental Footprint of Passenger Car Tires. J. Ind. Ecol..

[11] Mohajerani A., Burnett L., Smith J.V., Markovski S., Rodwell G., Rahman M.T., Kurmus H., Mirzababaei M., Arulrajah A., Horpibulsuk S., Maghool F. (2020). Recycling Waste Rubber Tyres in Construction Materials and Associated Environmental Considerations: A Review. Resour. Conserv. Recycl..

[12] Kole P.J., Löhr A.J., Van Belleghem F.G.A.J., Ragas A.M.J. (2017). Wear and Tear of Tyres: A Stealthy Source of Microplastics in the Environment. Int. J. Environ. Res. Public Health.

[13] Wagner S., Hüffer T., Klöckner P., Wehrhahn M., Hofmann T., Reemtsma T. (2018). Tire Wear Particles in the Aquatic Environment - A Review on Generation, Analysis, Occurrence, Fate and Effects. Water Res..

[14] Giechaskiel B., Grigoratos T., Mathissen M., Quik J., Tromp P., Gustafsson M., Franco V., Dilara P. (2024). Contribution of Road Vehicle Tyre Wear to Microplastics and Ambient Air Pollution. Sustainability.

[15] Kumar A., Dhanorkar R.J., Mohanty S., Gupta V.K. (2024). Advances in Recycling of Waste Vulcanized Rubber Products via Different Sustainable Approaches. Mater. Adv..

[16] Abdullah Z.T. (2024). Remanufactured Waste Tire By-Product Valorization: Quantitative–Qualitative Sustainability-Based Assessment. Results Eng..

[17] Zarmehr S.P., Pahlavan F., Kazemi M., Lamanna A., Fini E.H. (2026). Enabling Durable Circular Reuse of Sulfur in Asphalt by Revealing the Mechanisms of Photochemical and Moisture-Driven Degradation. J. Ind. Eng. Chem..

[18] Lostier A., Sarica T., Lasne J., Roose A., Sartelet K., Jamar M., Gaudion V., Dusanter S., Lesueur D., Chen H. (2025). Real-World Asphalt Pavement Emissions: Combining Simulation Chamber Measurements and City Scale Modeling to Elucidate the Impacts on Air Quality. ACS EST. Air.

[19] Bej P.K., Shariati S., Lostier A., Solaiman S., Chen H., Tomas A., Houzel N., Danjou P.-E., Coeur C., Romanias M.N., Fini E. (2026). VOC Emissions from Asphalt: Laboratory Oxidation, Ultrafine Particle Formation, and Urban Air Quality Implications. J. Hazard. Mater..

[20] Lee H., Ju M., Kim Y. (2020). Estimation of Emission of Tire Wear Particles (TWPs) in Korea. Waste Manag..

[21] Buadit T., Rattanapan C., Ussawarujikulchai A., Suchiva K., Papong S., Ma H.w. (2020). Life Cycle Assessment of Material Recovery from Pyrolysis Process of End-of-Life Tires in Thailand. Int. J. Environ. Sci. Dev..

[22] Abbas-Abadi M.S., Kusenberg M., Shirazi H.M., Goshayeshi B., Van Geem K.M. (2022). Towards Full Recyclability of End-of-Life Tires: Challenges and Opportunities. J. Clean. Prod..

[23] Vahdatbin M., Jamshidi M. (2022). Using Chemical Agent in Microwave Assisted Devulcanization of NR/SBR Blends: An Effective Recycling Method. Resour. Conserv. Recycl..

[24] Dobrotă D., Dobrotă G., Dobrescu T. (2020). Improvement of Waste Tyre Recycling Technology Based on a New Tyre Markings. J. Clean. Prod..

[25] Park B., Cho K., Choi K., Hong S.H. (2025). Catalytic and Selective Chemical Recycling of Post-Consumer Rubbers into Cycloalkenes. Chem.

[26] Putro W.S., Ueda Y., Yamashita H., Kamei N., Faried M., Hatori M., Hojo M., Tahara S., Homma M., Minato T. (2025). Two-Step Chemical Recycling of Tire Rubber to Isoprene. ACS Catal..

[27] Wiśniewska P., Haponiuk J.T., Colom X., Saeb M.R. (2023). Green Approaches in Rubber Recycling Technologies: Present Status and Future Perspective. ACS Sustain. Chem. Eng..

[28] Formela K. (2022). Waste Tire Rubber-Based Materials: Processing, Performance Properties and Development Strategies. Adv. Ind. Eng. Polym. Res..

[29] Chittella H., Yoon L.W., Ramarad S., Lai Z.W. (2021). Rubber Waste Management: A Review on Methods, Mechanism, and Prospects. Polym. Degrad. Stab..

[30] Dorigato A., Rigotti D., Fredi G. (2023). Recent Advances in the Devulcanization Technologies of Industrially Relevant Sulfur-Vulcanized Elastomers. Adv. Ind. Eng. Polym. Res..

[31] Asaro L., Gratton M., Seghar S., Aït Hocine N. (2018). Recycling of Rubber Wastes by Devulcanization. Resour. Conserv. Recycl..

[32] Huang Y., Bird R.N., Heidrich O. (2007). A Review of the Use of Recycled Solid Waste Materials in Asphalt Pavements. Resour. Conserv. Recycl..

[33] Zhou H., Holikatti S., Vacura P. (2014). Caltrans Use of Scrap Tires in Asphalt Rubber Products: A Comprehensive Review. J. Traffic Transport. Eng..

[34] Zou F., Leng Z., Li D., Zhang S., Yaphary Y.L., Lu G. (2023). Leaching Potential of Metals and PAHs of Asphalt Rubber Paving Materials. Transport. Res. Transport Environ..

[35] Vega B., Montero L., Lincoln S., Agulló N., Borrós S. (2008). Control of Vulcanizing/Devulcanizing Behavior of Diphenyl Disulfide with Microwaves as the Heating Source. J. Appl. Polym. Sci..

[36] de Sousa F.D.B., Zanchet A., Scuracchio C.H. (2019). From Devulcanization to Revulcanization: Challenges in Getting Recycled Tire Rubber for Technical Applications. ACS Sustain. Chem. Eng..

[37] Sathiskumar C., Karthikeyan S. (2019). Recycling of Waste Tires and Its Energy Storage Application of By-Products –a Review. Sustain. Mater. Technol..

[38] Gnanaraj J.S., Lee R.J., Levine A.M., Wistrom J.L., Wistrom S.L., Li Y., Li J., Akato K., Naskar A.K., Paranthaman M.P. (2018). Sustainable Waste Tire Derived Carbon Material as a Potential Anode for Lithium-Ion Batteries. Sustain. Times.

[39] Akhtar A.Y., Tsang H.H. (2024). Dynamic Leaching Assessment of Recycled Polyurethane-Coated Tire Rubber for Sustainable Engineering Applications. Chem. Eng. J..

[40] Wu Q., Zhang Q., Chen X., Song G., Xiao J. (2024). Life Cycle Assessment of Waste Tire Recycling: Upgraded Pyrolytic Products for New Tire Production. Sustain. Prod. Consum..

[41] Foley M. (2020).

[42] Goevert D. (2024). The Value of Different Recycling Technologies for Waste Rubber Tires in the Circular Economy—A Review. Front. Sustain..

[43] Simcox N.J., Bracker A., Ginsberg G., Toal B., Golembiewski B., Kurland T., Hedman C. (2011). Synthetic Turf Field Investigation in Connecticut. J. Toxicol. Environ. Health. A.

[44] Pavilonis B.T., Weisel C.P., Buckley B., Lioy P.J. (2014). Bioaccessibility and Risk of Exposure to Metals and SVOCs in Artificial Turf Field Fill Materials and Fibers. Risk Anal..

[45] Schneider K., de Hoogd M., Haxaire P., Philipps A., Bierwisch A., Kaiser E. (2020). ERASSTRI - European Risk Assessment Study on Synthetic Turf Rubber Infill – Part 2: Migration and Monitoring Studies. Sci. Total Environ..

[46] Roetman E., Joustra J., Heideman G., Balkenende R. (2024). Does the Rubber Meet the Road ? Assessing the Potential of Devulcanization Technologies for the Innovation of Tire Rubber Recycling. Sustainability.

[47] Wiśniewska P., Wang S., Formela K. (2022). Waste Tire Rubber Devulcanization Technologies: State-of-the-Art, Limitations and Future Perspectives. Waste Manag..

[48] Markl E., Lackner M. (2020). Devulcanization Technologies for Recycling of Tire-Derived Rubber: A Review. Materials.

[49] de Jesus A., Antunes P., Santos R., Mendonça S. (2019). Eco-Innovation Pathways to a Circular Economy: Envisioning Priorities through a Delphi Approach. J. Clean. Prod..

[50] Higashiyama H., Sano M., Nakanishi F., Takahashi O., Tsukuma S. (2016). Field Measurements of Road Surface Temperature of Several Asphalt Pavements with Temperature Rise Reducing Function. Case Stud. Constr. Mater..

[51] Hashamfirooz M., Dehghani M.H., Khanizadeh M., Aghaei M., Bashardoost P., Hassanvand M.S., Hassanabadi M., Momeniha F. (2025). A Systematic Review of the Environmental and Health Effects of Waste Tires Recycling. Heliyon.

[52] Sibeko M.A., Adeniji A.O., Okoh O.O., Hlangothi S.P. (2020). Trends in the Management of Waste Tyres and Recent Experimental Approaches in the Analysis of Polycyclic Aromatic Hydrocarbons (PAHs) from Rubber Crumbs. Environ. Sci. Pollut. Res. Int..

[53] Grynkiewicz-Bylina B., Rakwic B., Słomka-Słupik B. (2022). Tests of Rubber Granules Used as Artificial Turf for Football Fields in Terms of Toxicity to Human Health and the Environment. Sci. Rep..

[54] Ruffino B., Fiore S., Zanetti M.C. (2013). Environmental-Sanitary Risk Analysis Procedure Applied to Artificial Turf Sports Fields. Environ. Sci. Pollut. Res. Int..

[55] Zhou X., Zhai Y., Zhang T., Li Z., Cheng Z., Li C., Xu T., Hong J. (2023). Uncovering the Energy-Carbon-Water Footprint of Waste Rubber Recycling: Integrated Environmental and Economic Perspectives. J. Environ. Manage..

[56] Farina A., Ruffino B., Kutay E., Anctil A. (2024). Leaching Behavior of Metals from Asphalt Mixtures Modified with Crumb Rubber from Scrap Tires. Waste Manag..

[57] Li L., Zhou T., Cao L., Zhou J., Liu Z., Dong Z. (2024). Characterization of Emissions from Rubber Modified Asphalt and Their Impact on Environmental Burden: Insights into Composition Variability and Hazard Assessment. J. Hazard. Mater..

[58] Mousavi M., Aldagari S., Fini E.H. (2023). Adsorbing Volatile Organic Compounds within Bitumen Improves Colloidal Stability and Air Quality. ACS Sustain. Chem. Eng..

[59] Yu H., Leng Z., Zhou Z., Shih K., Xiao F., Gao Z. (2017). Optimization of Preparation Procedure of Liquid Warm Mix Additive Modified Asphalt Rubber. J. Clean. Prod..

[60] Leng Z., Yu H., Zhang Z., Tan Z. (2017). Optimizing the Mixing Procedure of Warm Asphalt Rubber with Wax-Based Additives through Mechanism Investigation and Performance Characterization. Constr. Build. Mater..

[61] Li D., Leng Z., Zou F., Yu H. (2021). Effects of Rubber Absorption on the Aging Resistance of Hot and Warm Asphalt Rubber Binders Prepared with Waste Tire Rubber. J. Clean. Prod..

[62] Perkins A.N., Inayat-Hussain S.H., Deziel N.C., Johnson C.H., Ferguson S.S., Garcia-Milian R., Thompson D.C., Vasiliou V. (2019). Evaluation of Potential Carcinogenicity of Organic Chemicals in Synthetic Turf Crumb Rubber. Environ. Res..

[63] Mohajerani A., Kurmus H., Conti D., Cash L., Semcesen A., Abdurahman M., Rahman M.T. (2022). Environmental Impacts and Leachate Analysis of Waste Rubber Incorporated in Construction and Road Materials: A Review. Sci. Total Environ..

[64] Pochron S.T., Fiorenza A., Sperl C., Ledda B., Lawrence Patterson C., Tucker C.C., Tucker W., Ho Y.L., Panico N. (2017). The Response of Earthworms (Eisenia Fetida) and Soil Microbes to the Crumb Rubber Material Used in Artificial Turf Fields. Chemosphere.

[65] Lu F., Su Y., Ji Y., Ji R. (2021). Release of Zinc and Polycyclic Aromatic Hydrocarbons From Tire Crumb Rubber and Toxicity of Leachate to Daphnia Magna: Effects of Tire Source and Photoaging. Bull. Environ. Contam. Toxicol..

[66] Celeiro M., Dagnac T., Llompart M. (2018). Determination of Priority and Other Hazardous Substances in Football Fields of Synthetic Turf by Gas Chromatography-Mass Spectrometry: A Health and Environmental Concern. Chemosphere.

[67] Llompart M., Sanchez-Prado L., Pablo Lamas J., Garcia-Jares C., Roca E., Dagnac T. (2013). Hazardous Organic Chemicals in Rubber Recycled Tire Playgrounds and Pavers. Chemosphere.

[68] Sheng Y., Liu Y., Wang K., Cizdziel J.V., Wu Y., Zhou Y. (2021). Ecotoxicological Effects of Micronized Car Tire Wear Particles and Their Heavy Metals on the Earthworm (Eisenia Fetida) in Soil. Sci. Total Environ..

[69] Hennequin T., van Vlimmeren L., Mostoni S., Pomilla F.R., Scotti R., Stauch C., van der Hulst M.K., Huijbregts M.A.J., van Zelm R. (2024). Environmental Impact Prediction of a New Tire Vulcanization Activator. ACS Sustain. Chem. Eng..

[70] Shalaby A., Khan R.A. (2005). Design of Unsurfaced Roads Constructed with Large-Size Shredded Rubber Tires: A Case Study. Resour. Conserv. Recycl..

[71] Azizian M.F., Nelson P.O., Thayumanavan P., Williamson K.J. (2003). Environmental Impact of Highway Construction and Repair Materials on Surface and Ground Waters. Waste Manag..

[72] Kalbe U., Krüger O., Wachtendorf V., Berger W., Hally S. (2013). Development of Leaching Procedures for Synthetic Turf Systems Containing Scrap Tyre Granules. Waste Biomass Valorization.

[73] Formela K. (2021). Sustainable Development of Waste Tires Recycling Technologies – Recent Advances, Challenges and Future Trends. Adv. Ind. Eng. Polym. Res..

[74] Johannessen C., Liggio J., Zhang X., Saini A., Harner T. (2022). Composition and Transformation Chemistry of Tire-Wear Derived Organic Chemicals and Implications for Air Pollution. Atmos. Pollut. Res..

[75] Gągol M., Boczkaj G., Haponiuk J., Formela K. (2015). Investigation of Volatile Low Molecular Weight Compounds Formed during Continuous Reclaiming of Ground Tire Rubber. Polym. Degrad. Stab..

[76] Tandon V., Velazco D.A., Nazarian S., Picornell M. (2007). Performance Monitoring of Embankments Containing Tire Chips: Case Study. J. Perform. Constr. Facil..

[77] Zedler Ł., Kowalkowska-Zedler D., Vahabi H., Saeb M.R., Colom X., Cañavate J., Wang S., Formela K. (2019). Preliminary Investigation on Auto-Thermal Extrusion of Ground Tire Rubber. Materials.

[78] Formela K. (2022). Analysis of Volatile Organic Compounds Emission in the Rubber Recycling Products Quality Assessment. Adv. Ind. Eng. Polym. Res..

[79] Hejna A., Kosmela P., Olszewski A., Zedler Ł., Formela K., Skórczewska K., Piasecki A., Marć M., Barczewski R., Barczewski M. (2024). Management of Ground Tire Rubber Waste by Incorporation into Polyurethane-Based Composite Foams. Environ. Sci. Pollut. Res. Int..

[80] Susa D., Haydary J. (2013). Sulphur Distribution in the Products of Waste Tire Pyrolysis. Chem. Pap..

[81] Zhang X., Tang J., Chen J. (2022). Behavior of Sulfur during Pyrolysis of Waste Tires: A Critical Review. J. Energy Inst..

[82] Hu H., Fang Y., Liu H., Yu R., Luo G., Liu W., Li A., Yao H. (2014). The Fate of Sulfur during Rapid Pyrolysis of Scrap Tires. Chemosphere.

[83] Zhang J., Zhu M., Jones I., Okoye C.O., Zhang Z., Zhang D. (2021). The Transformation and Fate of Sulphur during CO2 Gasification of a Spent Tyre Pyrolysis Char. Proc. Combust. Inst..

[84] Jones I., Zhu M., Zhang J., Zhang Z., Preciado-Hernandez J., Gao J., Zhang D. (2021). The Application of Spent Tyre Activated Carbons as Low-Cost Environmental Pollution Adsorbents: A Technical Review. J. Clean. Prod..

[85] Mentes D., Tóth C.E., Nagy G., Muránszky G., Póliska C. (2022). Investigation of Gaseous and Solid Pollutants Emitted from Waste Tire Combustion at Different Temperatures. Waste Manag..

[86] Jeong Y., Lee S., Woo S.-H. (2022). Chemical Leaching from Tire Wear Particles with Various Treadwear Ratings. Int. J. Environ. Res. Public Health.

[87] Selbes M., Yilmaz O., Khan A.A., Karanfil T. (2015). Leaching of DOC, DN, and Inorganic Constituents from Scrap Tires. Chemosphere.

[88] Duda A., Kida M., Ziembowicz S., Koszelnik P. (2020). Application of Material from Used Car Tyres in Geotechnics—an Environmental Impact Analysis. PeerJ.

[89] Hennebert P., Lambert S., Fouillen F., Charrasse B. (2014). Assessing the Environmental Impact of Shredded Tires as Embankment Fill Material. Can. Geotech. J..

[90] Abdul Sattar M. (2024). Curcuma Longa-Based Green and Sustainable Antioxidants for Biopolymers. ACS Sustain. Chem. Eng..

[91] Chung J.-Y., Hwang U., Kim J., Kim N.-Y., Nam J., Jung J., Kim S.-H., Cho J.K., Lee B., Park I.-K. (2023). Amine-Functionalized Lignin as an Eco-Friendly Antioxidant for Rubber Compounds. ACS Sustain. Chem. Eng..

[92] Du B., Liang B., Li Y., Shen M., Liu L.-Y., Zeng L. (2022). First Report on the Occurrence of N -(1,3-Dimethylbutyl)- N ′-Phenyl- p -Phenylenediamine (6PPD) and 6PPD-Quinone as Pervasive Pollutants in Human Urine from South China. Environ. Sci. Technol. Lett..

[93] Hu X., Zhao H.N., Tian Z., Peter K.T., Dodd M.C., Kolodziej E.P. (2022). Transformation Product Formation upon Heterogeneous Ozonation of the Tire Rubber Antioxidant 6PPD (N-(1,3dimethylbutyl)-N′-Phenyl-p-Phenylenediamine). Environ. Sci. Technol. Lett..

[94] Varshney S., Gora A.H., Siriyappagouder P., Kiron V., Olsvik P.A. (2022). Toxicological Effects of 6PPD and 6PPD Quinone in Zebrafish Larvae. J. Hazard. Mater..

[95] Johannessen C., Helm P., Lashuk B., Yargeau V., Metcalfe C.D. (2022). The Tire Wear Compounds 6PPD-Quinone and 1,3-Diphenylguanidine in an Urban Watershed. Arch. Environ. Contam. Toxicol..

[96] Chang J., Zhang L., Zhao J., Zhang Z., Wang Z., Wang H., Wan B. (2024). 6PPD, Not 6PPD-Quinone, Induced Serious Zebrafish Eye Damage by Disrupting the Thyroid Signaling Pathway. Environ. Sci. Technol..

[97] Scholz N.L., Myers M.S., McCarthy S.G., Labenia J.S., McIntyre J.K., Ylitalo G.M., Rhodes L.D., Laetz C.A., Stehr C.M., French B.L. (2011). Recurrent Die-Offs of Adult Coho Salmon Returning to Spawn in Puget Sound Lowland Urban Streams. PLoS One.

[98] Jiang Y., Wang C., Ma L., Gao T., Wāng Y. (2024). Environmental Profiles, Hazard Identification, and Toxicological Hallmarks of Emerging Tire Rubber-Related Contaminants 6PPD and 6PPD-Quinone. Environ. Int..

[99] Deng M., Ji X., Peng B., Fang M. (2024). In Vitro and In Vivo Biotransformation Profiling of 6PPD-Quinone toward Their Detection in Human Urine. Environ. Sci. Technol..

[100] Helm P.A., Raby M., Kleywegt S., Sorichetti R.J., Arabian G., Smith D., Howell E.T., Thibeau J. (2024). Assessment of Tire-Additive Transformation Product 6PPD-Quinone in Urban-Impacted Watersheds. ACS ES&T Water.

[101] Chen X., He T., Yang X., Gan Y., Qing X., Wang J., Huang Y. (2023). Analysis, Environmental Occurrence, Fate and Potential Toxicity of Tire Wear Compounds 6PPD and 6PPD-Quinone. J. Hazard. Mater..

[102] Papan D. (2024).

[103] Zhao H.N., Thomas S.P., Zylka M.J., Dorrestein P.C., Hu W. (2023). Urine Excretion, Organ Distribution, and Placental Transfer of 6PPD and 6PPD-Quinone in Mice and Potential Developmental Toxicity through Nuclear Receptor Pathways. Environ. Sci. Technol..

[104] Zoroufchi Benis K., Behnami A., Minaei S., Brinkmann M., McPhedran K.N., Soltan J. (2023). Environmental Occurrence and Toxicity of 6PPD Quinone, an Emerging Tire Rubber-Derived Chemical: A Review. Environ. Sci. Technol. Lett..

[105] Ji J., Li C., Zhang B., Wu W., Wang J., Zhu J., Liu D., Gao R., Ma Y., Pang S., Li X. (2022). Exploration of Emerging Environmental Pollutants 6PPD and 6PPDQ in Honey and Fish Samples. Food Chem..

[106] Song S., Gao Y., Feng S., Cheng Z., Huang H., Xue J., Zhang T., Sun H. (2024). Widespread Occurrence of Two Typical N, N’-Substituted p-Phenylenediamines and Their Quinones in Humans: Association with Oxidative Stress and Liver Damage. J. Hazard. Mater..

[107] Montgomery D., Ji X., Cantin J., Philibert D., Foster G., Selinger S., Jain N., Miller J., McIntyre J., de Jourdan B. (2023). Interspecies Differences in 6PPD-Quinone Toxicity Across Seven Fish Species: Metabolite Identification and Semiquantification. Environ. Sci. Technol..

[108] Grasse N., Seiwert B., Massei R., Scholz S., Fu Q., Reemtsma T. (2023). Uptake and Biotransformation of the Tire Rubber-Derived Contaminants 6-PPD and 6-PPD Quinone in the Zebrafish Embryo ( Danio Rerio ). Environ. Sci. Technol..

[109] Peng B., Liu M., Han Y., Wanjaya E.R., Fang M. (2019). Competitive Biotransformation Among Phenolic Xenobiotic Mixtures: Underestimated Risks for Toxicity Assessment. Environ. Sci. Technol..

[110] Lokesh S., Arunthavabalan S., Hajj E., Hitti E., Yang Y. (2023). Investigation of 6PPD-Quinone in Rubberized Asphalt Concrete Mixtures. ACS Environ. Au.

[111] Wagner S., Klöckner P., Reemtsma T. (2022). Aging of Tire and Road Wear Particles in Terrestrial and Freshwater Environments – A Review on Processes, Testing, Analysis and Impact. Chemosphere.

[112] Jiang W., Gan J., Haver D. (2011). Sorption and Desorption of Pyrethroid Insecticide Permethrin on Concrete. Environ. Sci. Technol..

[113] Bhalode P., Najmi S., Vlachos D. (2024). Computation-Guided Removal of 6PPD from End-of-Life Waste Tires. ACS Sustain. Resour. Manag..

[114] Baensch-Baltruschat B., Kocher B., Kochleus C., Stock F., Reifferscheid G. (2021). Tyre and Road Wear Particles - A Calculation of Generation, Transport and Release to Water and Soil with Special Regard to German Roads. Sci. Total Environ..

[115] Halle L.L., Palmqvist A., Kampmann K., Khan F.R. (2020). Ecotoxicology of Micronized Tire Rubber: Past, Present and Future Considerations. Sci. Total Environ..

[116] Halsband C., Sørensen L., Booth A.M., Herzke D. (2020). Car Tire Crumb Rubber: Does Leaching Produce a Toxic Chemical Cocktail in Coastal Marine Systems?. Front. Environ. Sci..

[117] Kim J., Yang S.I., Moon H., Hong J., Hong J., Choi W., Son H., Lee B.c., Kim G.B., Kim Y. (2021). Potential Release of Nano-Carbon Black from Tire-Wear Particles through the Weathering Effect. J. Ind. Eng. Chem..

[118] Fiksel J., Bakshi B.R., Baral A., Guerra E., DeQuervain B. (2011). Comparative Life Cycle Assessment of Beneficial Applications for Scrap Tires. Clean Technol. Environ. Policy.

[119] Magnusson S., Mácsik J. (2017). Analysis of Energy Use and Emissions of Greenhouse Gases, Metals and Organic Substances from Construction Materials Used for Artificial Turf. Resour. Conserv. Recycl..

[120] Piao Z., Bueno M., Poulikakos L.D., Hellweg S. (2022). Life Cycle Assessment of Rubberized Semi-Dense Asphalt Pavements; A Hybrid Comparative Approach. Resour. Conserv. Recycl..

[121] Passarini F., Neri E., Berti B., Vassura I., Giorgini L., Zattini G., Tosi C., Cavazzoni M. (2018). Application of Lca Methodology in the Assessment of a Pyrolysis Process for Tyres Recycling. Environ. Eng. Manag. J..

[122] Morandi P., Folkersma R., Loos K., Voet V.S.D. (2026). Dynamic Covalent Polymer Networks Based on Renewable Feedstocks: A Perspective on the Role of Vitrimers in a Closed-Loop Economy. Biomacromolecules.

[123] Ren T., Yue X., Formela K., Rodrigue D., Colom Fajula X., McNally T., Dawei D., Zhang Y., Wang S. (2025). Progress in Devulcanization of Waste Tire Rubber: Upcycling towards a Circular Economy. Express Polym. Lett..

[124] Wiśniewska P., Ezzati P., Haponiuk J., Hejna A., Colom X., Saeb M.R. (2025). Towards Developing Fully Sustainable Elastomers: The Role of Chemistry. Green Chem..

[125] Adhikari B., De D., Maiti S. (2000). Reclamation and Recycling of Waste Rubber. Prog. Polym. Sci..

[126] Simon D.Á., Bárány T. (2023). Microwave Devulcanization of Ground Tire Rubber and Its Improved Utilization in Natural Rubber Compounds. ACS Sustain. Chem. Eng..

[127] Toteva V., Stanulov K. (2020). Waste Tires Pyrolysis Oil as a Source of Energy: Methods for Refining. Prog. Rubber Plast. Recycl. Technol..

[128] Banala D., Sabri Y., Choudhury N.R., Parthasarathy R. (2025). Sustainable Valorisation of End-of-Life Tyres Through Pyrolysis-Derived Recovered Carbon Black in Polymer Composites. Polymers.

[129] Schwirn K., Völker D., Løfstedt M., Fantke P., Bossa C., Sharma A., Posthuma L., Karakoltzidis A., Nikiforou F., Mikołajczyk A. (2025). The European Commission’s Safe and Sustainable by Design Framework: Bridging Innovation and Legislation. Environ. Sci. Eur..

[130] Tamborski M., Rojek I., Mikołajewski D. (2023). Revolutionizing Tire Quality Control: AI’s Impact on Research, Development, and Real-Life Applications. Appl. Sci..

[131] Luo Z., Wang H., Wang Z., Zhang X., Yan C., Yu R., Zhang H., Hu G., Xing B. (2024). Tire and Road Wear Particles Contribute Highly to N-(1,3-Dimethylbutyl)-N′ -Phenyl-p-Phenylenediamine (6PPD) and Heavy Metals in Road Dust on Driving School Grounds and Their Risk Implications in China. Environ. Technol. Innov..

[132] Lin J.-H., Wang S.-B. (2017). An Effective Route to Transform Scrap Tire Carbons into Highly-Pure Activated Carbons with a High Adsorption Capacity of Ethylene Blue through Thermal and Chemical Treatments. Environ. Technol. Innov..

